# Gene-Based Analysis of Regionally Enriched Cortical Genes in GWAS Data Sets of Cognitive Traits and Psychiatric Disorders

**DOI:** 10.1371/journal.pone.0031687

**Published:** 2012-02-22

**Authors:** Kari M. Ersland, Andrea Christoforou, Christine Stansberg, Thomas Espeseth, Manuel Mattheisen, Morten Mattingsdal, Gudmundur A. Hardarson, Thomas Hansen, Carla P. D. Fernandes, Sudheer Giddaluru, René Breuer, Jana Strohmaier, Srdjan Djurovic, Markus M. Nöthen, Marcella Rietschel, Astri J. Lundervold, Thomas Werge, Sven Cichon, Ole A. Andreassen, Ivar Reinvang, Vidar M. Steen, Stephanie Le Hellard

**Affiliations:** 1 Dr E. Martens Research Group for Biological Psychiatry, Department of Clinical Medicine, University of Bergen, Bergen, Norway; 2 Center for Medical Genetics and Molecular Medicine, Haukeland University Hospital, Bergen, Norway; 3 Department of Psychology, Center for the Study of Human Cognition, University of Oslo, Oslo, Norway; 4 Department of Genomics, Life & Brain Center, University of Bonn, Bonn, Germany; 5 Department of Biostatistics, Harvard School of Public Health, Boston, Massachusetts, United States of America; 6 Institute for Medical Biometry, Informatics, and Epidemiology (IMBIE), University of Bonn, Bonn, Germany; 7 Institute of Clinical Medicine, University of Oslo, Oslo, Norway; 8 Sørlandet Hospital HF, Kristiansand, Norway; 9 Research Institute of Biological Psychiatry, Copenhagen University Hospital, Roskilde, Denmark; 10 Department of Genetic Epidemiology in Psychiatry, Central Institute of Mental Health, University of Mannheim, Mannheim, Germany; 11 Department of Medical Genetics, Oslo University Hospital, Ullevål, Oslo, Norway; 12 Institute of Human Genetics, University of Bonn, Bonn, Germany; 13 German Center for Neurodegenerative Disorders (DZNE), Bonn, Germany; 14 Department of Biological and Medical Psychology, University of Bergen, Bergen, Norway; 15 Kavli Research Centre for Aging and Dementia, Haraldsplass Deaconesses Hospital, Bergen, Norway; 16 Institute of Neuroscience and Medicine (INM-1), Research Center Juelich, Juelich, Germany; 17 Division of Mental Health and Addiction, Oslo University Hospital, Oslo, Norway; University of Illinois-Chicago, United States of America

## Abstract

**Background:**

Despite its estimated high heritability, the genetic architecture leading to differences in cognitive performance remains poorly understood. Different cortical regions play important roles in normal cognitive functioning and impairment. Recently, we reported on sets of regionally enriched genes in three different cortical areas (frontomedial, temporal and occipital cortices) of the adult rat brain. It has been suggested that genes preferentially, or specifically, expressed in one region or organ reflect functional specialisation. Employing a gene-based approach to the analysis, we used the regionally enriched cortical genes to mine a genome-wide association study (GWAS) of the Norwegian Cognitive NeuroGenetics (NCNG) sample of healthy adults for association to nine psychometric tests measures. In addition, we explored GWAS data sets for the serious psychiatric disorders schizophrenia (SCZ) (n = 3 samples) and bipolar affective disorder (BP) (n = 3 samples), to which cognitive impairment is linked.

**Principal Findings:**

At the single gene level, the temporal cortex enriched gene *RAR-related orphan receptor B* (*RORB*) showed the strongest overall association, namely to a test of verbal intelligence (Vocabulary, *P* = 7.7E-04). We also applied gene set enrichment analysis (GSEA) to test the candidate genes, as gene sets, for enrichment of association signal in the NCNG GWAS and in GWASs of BP and of SCZ. We found that genes differentially expressed in the temporal cortex showed a significant enrichment of association signal in a test measure of non-verbal intelligence (Reasoning) in the NCNG sample.

**Conclusion:**

Our gene-based approach suggests that *RORB* could be involved in verbal intelligence differences, while the genes enriched in the temporal cortex might be important to intellectual functions as measured by a test of reasoning in the healthy population. These findings warrant further replication in independent samples on cognitive traits.

## Introduction

Cognitive abilities (e.g. intelligence, memory, attention and speed of processing) vary to a great extent in the population, considerably affecting the life outcome of individuals. Despite being highly heritable, with estimates ranging from 30–80%, little is known about the genetic mechanisms involved in cognitive functioning (reviewed in [1]). It is, however, widely accepted that a polygenic mechanism underlies the differences in cognition, each genetic factor having a very small effect size (reviewed in [1], [2]). A recent genome-wide association study (GWAS) showed, for the first time, that common genetic variants account for ∼40–50% of the variation in human intelligence [3]. However, despite an extensive search by linkage and association studies, only a limited number of genes has so far been implicated in normal cognitive functioning (e.g. *ALDH5A1*, *APOE*, *COMT*, *BDNF*, *DCLK1*) [1], [4]–[9].

Cognitive dysfunction is one of the main clinical problems observed in patients suffering from major psychiatric disorders, such as schizophrenia (SCZ) and bipolar affective disorder (BP). High heritability has been estimated for both SCZ and BP [10]–[12], and common alleles of small effect are thought to increase susceptibility to these complex disorders. However, for SCZ, some rare variants (e.g. copy number variations) have also been linked to disease susceptibility [13], [14]. Although great efforts have been made over the last decades to identify genetic factors causing susceptibility to SCZ and BP, surprisingly few genes have so far been implicated [15]–[22]. By considering cognition as an intermediate biological phenotype (endophenotype) for major psychiatric illnesses, one might come closer to identifying causative genetic factors. An overlap in genetic factors linked to both cognition and psychiatric disorders has already been observed (e.g. *ZNF804A* and *DISC1*) [18], [23], which supports the validity of testing the same genes in both normal cognitive function and in psychiatric illnesses.

Several areas of the brain, in particular different cortical regions, play important roles in normal cognitive functioning and impairment, as well as in psychiatric disease. A network consisting of areas in the dorsolateral prefrontal, parietal, anterior cingulate, temporal and occipital cortices (parieto-frontal integration theory) has been associated with differences in intellectual function [24]. The prefrontal cortex is particularly important for working memory, attention and planning, and structural and functional changes in this region have been linked to psychiatric disorders. Regions within the temporal and occipital lobes have also been implicated in cognitive abilities and psychiatric disorders, as these regions are critical for early auditory and visual sensory information processing and interpretation. In general, reduction of cortical thickness has been observed in patients suffering from SCZ and BP, particularly in the frontal and temporal lobes [25], while total brain volume (gray and white matter) and cortical thickness have been correlated to measures of intelligence [26], [27].

Previously, we examined the global gene expression in the frontomedial (FMCx), temporal (TCx) and occipital (OCx) cortices from the normal adult rat brain, and identified distinct sets of regionally enriched cortical genes [28], [29]. While the overall gene expression in the different cortical areas was highly similar, 65 genes showed marked regional enrichment (30, 24 and 11 genes in the FMCx, TCx and OCx, respectively). Based upon the assumption that genes highly or specifically expressed within a certain region or organ are likely to reflect its functional specialisation [28], [30], [31], and considering the implications of different areas of the cortex in human cognition and psychiatric disorders, we hypothesised that these enriched genes might serve as candidates for individual differences in cognitive function and for psychiatric disorders.

In this study, we used the regionally enriched cortical genes as candidates to mine existing GWASs of relevant cognitive traits and of SCZ and BP, taking a gene-based approach. First, we applied a novel tool, LDsnpR, (Christoforou *et al.* under revision) to assign single nucleotide polymorphism (SNP) marker information from the GWAS data to their corresponding genes, and then to subsequently score the genes. Applying this gene-based approach, we tested the association of regionally enriched cortical genes to normal cognitive functioning using a GWAS recently conducted by our group (Christoforou *et al.* unpublished data). Next, we analysed these genes, as gene sets, using gene set enrichment analysis (GSEA) [32] to search for enrichment of association signal in the aforementioned GWAS of cognition and in GWASs of psychiatric disorders (SCZ and BP).

## Materials and Methods

### Candidate genes

#### Selection of candidate genes

Recently, we described sets of genes that show differential expression in three different cortical regions in the adult rat brain (FMCx, TCx and OCx) [29], based on global gene expression analysis of several brain regions (three cortical regions, as well as hippocampus, striatum and cerebellum) and three non-CNS samples (liver, kidney and spleen) [28]. Sixty-five genes were found to display enriched expression in certain cortical regions (30, 24 and 11 genes in the FMCx, TCx and OCx, respectively) [29]. The Ensembl Genome Browser (release 54) was searched to identify the Ensembl ID for the human homologues to the rat genes (http://may2009.archive.ensembl.org/) [33]. Three genes were not represented in the Ensembl release 54 (i.e. two unassigned Celera genes: rCG46329 and rCG41008; and Clec2l), resulting in 62 genes eligible for the subsequent gene-based analysis in cognition and psychiatric disorders (Table 1–3).

**Table 1:** Overview of frontomedial cortex enriched genes analysed in this study.

HGNC Symbol	Ensembl ID/54	Description
ADPRHL1	ENSG00000153531	ADP-ribosylhydrolase like 1
ADRA1B	ENSG00000170214	Adrenergic receptor, alpha 1b
ALDH3B2	ENSG00000132746	Aldehyde dehydrogenase 3 family, member B2
C1QL3	ENSG00000165985	Complement component 1, q subcomponent-like 3
CADM1	ENSG00000182985	Cell adhesion molecule 1
CRIM1	ENSG00000150938	Cysteine rich transmembrane BMP regulator 1 (chordin like)
CRIP2	ENSG00000182809	Cysteine-rich protein 2
EFNB3	ENSG00000108947	Ephrin B3
EPHB6	ENSG00000106123	Eph receptor B6
FXYD6	ENSG00000137726	FXYD domain-containing ion transport regulator 6
GRP	ENSG00000134443	Gastrin releasing peptide
HAP1	ENSG00000173805	Huntingtin-associated protein 1, transcript variant 2.
HCRTR1	ENSG00000121764	Hypocretin (orexin) receptor 1
HEBP1	ENSG00000013583	Heme binding protein 1
LDB2	ENSG00000169744	LIM domain binding 2
LMO4	ENSG00000143013	LIM domain only 4
NAGS	ENSG00000161653	N-acetylglutamate synthase
NTF3	ENSG00000185652	Neurotrophin 3
PANX1	ENSG00000110218	Pannexin 1
PCDH17	ENSG00000118946	Protocadherin 17
PFKL	ENSG00000141959	Phosphofructokinase, liver, B-type
PRKCDBP	ENSG00000170955	Protein kinase C, delta binding protein
PRMT2	ENSG00000160310	Protein arginine N-methyltransferase 2
RSPO2	ENSG00000147655	R-spondin 2 homolog (Xenopus laevis)
RYR1	ENSG00000196218	Ryanodine receptor 1, skeletal muscle
ST6GALNAC5	ENSG00000117069	Sialyltransferase 7E
SULF2	ENSG00000196562	Sulfatase 2
TMEFF1	ENSG00000066697	Tomoregulin-1 Precursor (Transmembrane protein with EGF-like and one follistatin-like domain)(TR-1)
ZCCHC12	ENSG00000174460	Zinc finger, CCHC domain containing 12

The 29 frontomedial enriched cortical genes [29] were used as candidates to search for association to nine test measures of cognitive functions [37]–[40], at the single gene- and gene set-based level. The HUGO Gene Nomenclature Committee (HGNC) symbol, Ensembl Genome Browser (release 54) identification [33] and gene description is shown.

**Table 2 pone-0031687-t002:** Overview of temporal cortex enriched genes analysed in this study.

HGNC Symbol	Ensembl ID/54	Description
ARHGAP9	ENSG00000123329	Rho GTPase activating protein 9
ATOH7	ENSG00000179774	Atonal homolog 7 (Drosophila)
C1orf146	ENSG00000203910	Uncharacterized protein C1orf146
CA4	ENSG00000167434	Carbonic anhydrase 4
CABP1	ENSG00000157782	Calcium binding protein 1
CADPS2	ENSG00000081803	Ca2+-dependent activator for secretion protein 2
CD200R1	ENSG00000163606	CD200 receptor 1
COL13A1	ENSG00000197467	Collagen type XIII alpha-1 chain
GPR88	ENSG00000181656	G-protein coupled receptor 88
HHATL	ENSG00000010282	Hedgehog acyltransferase-like
IKBKE	ENSG00000143466	Inhibitor of kappaB kinase epsilon
JDP2	ENSG00000140044	Jun dimerization protein 2
KCNC1	ENSG00000129159	Potassium voltage gated channel, Shaw-related subfamily, member 1
KCNS1	ENSG00000124134	K+ voltage-gated channel, subfamily S, 1
LPHN2	ENSG00000117114	Latrophilin 2
LXN	ENSG00000079257	Latexin
NEFM	ENSG00000104722	Neurofilament, medium polypeptide
NEU2	ENSG00000115488	Sialidase 2 (cytosolic sialidase)
PLK5P	ENSG00000185988	Plk5 polo-like kinase 5
RORB	ENSG00000198963	RAR-related orphan receptor beta
SCN1A	ENSG00000144285	Sodium channel, voltage-gated, type 1, alpha polypeptide
SCN4B	ENSG00000177098	Sodium channel, voltage-gated, type IV, beta

The 22 temporal cortex enriched genes [29] were used as candidates to search for association to nine test measures of cognitive functions [37]–[40], at the single gene- and gene set-based level. The HUGO Gene Nomenclature Committee (HGNC) symbol, Ensembl Genome Browser (release 54) identification [33] and gene description is shown.

**Table 3:** Overview of occipital cortex enriched genes analysed in this study.

HGNC Symbol	Ensembl ID/54	Description
DCN	ENSG00000011465	Decorin
GPR68	ENSG00000119714	G protein-coupled receptor 68
HTR5B	ENSG00000125631	5-hydroxytryptamine (serotonin) receptor 5B
HTRA4	ENSG00000169495	Serine peptidase 4
IL12A	ENSG00000168811	Interleukin 12a
IRF6	ENSG00000117595	Interferon regulatory factor 6
KLF5	ENSG00000102554	Kruppel-like factor 5
MAB21L1	ENSG00000180660	Mab-21-like 1 (C. elegans)
NR2F1	ENSG00000175745	Nuclear receptor subfamily 2, group F, member 1 (Nr2f1).
ODZ3	ENSG00000218336	Odd Oz/ten-m homolog 3 (Drosophila)
SATB1	ENSG00000182568	SATB homeobox 1

The 11 occipital cortex enriched genes [29] were used as candidates to search for association to nine test measures of cognitive functions [37]–[40], at the single gene- and gene set-based level. The HUGO Gene Nomenclature Committee (HGNC) symbol, Ensembl Genome Browser (release 54) identification [33] and gene description is shown.

#### Expression and functional characterisation of candidate genes

The expression pattern of the human homologues to the rat genes were analysed in the Allen Human Cortex Study (Whole Brain Microarray Survey) from The Allen Institute for Brain Science [34] (http://humancortex.alleninstitute.org). Functional characterisation of the human homologous genes was performed using the Panther Classification System version 7 (http://www.pantherdb.org/) [35], [36], as previously described [28]. One gene was not represented in Panther (i.e. *HTR5B*).

### GWAS datasets

#### GWAS of cognition in the Norwegian Cognitive NeuroGenetics sample

The Norwegian Cognitive NeuroGenetics (NCNG) sample consists of 670 healthy adult individuals of Norwegian origin (214 males, 456 females), extensively tested for cognitive abilities. The participants were between 18 to 79 years of age (mean: 47.6), and were recruited through advertisements in local newspapers to participate at the University of Bergen (n = 171) and Oslo (n = 499) areas. In this study we focused on nine different tests, covering four major cognitive functions, namely: Intellectual function (The Vocabulary and Matrix Reasoning sub-tests from the Wechsler Abbreviated Scale of Intelligence, and the estimated Full-Scale Intelligence Quotient (FSIQ) [37]), memory (the total numbers of words learned across five trials (CVLT-L) and the delayed free recall score (CVLT-DR) from the California Verbal Learning Test [38]), executive attention (the third condition from the D-KEFS Color-Word Interference Test (Stroop3) [39]) and attention (Cued Discrimination Task, CDT-Valid, CDT-Invalid and CDT-Neutral [40]) (Table S1). Correlation estimates between the psychometric tests are listed in Table S2. The individuals were genotyped using the Illumina platform (Human610-Quad), and after quality control, 554,225 SNPs were incorporated into the analysis. Further details on the sample, genotyping and quality control can be found in Davies *et al.* 2011 [3].

#### GWAS of BP and SCZ

We mined the following GWAS data sets: for BP, we analysed the Norwegian Thematically Organized Psychosis (TOP) Study BP sample (198 cases and 336 controls, genotyped using the Affymetrix Genome-Wide Human SNP Array 6.0) [41], the British Wellcome Trust Case Control Consortium BP (WTCCC, 1,868 cases and 2,938 controls, genotyped using Affymetrix GC500K) [42] and a German BP GWAS (Bonn/Mannheim, 682 cases and 1,300 controls, genotyped using Illumina's HumanHap550v3) [20]: for SCZ, we mined GWAS data from the Norwegian TOP SCZ (201 cases and 305 controls, genotyped using Affymetrix Genome-Wide Human SNP Array 6.0) [43], the German part of a combined German-Dutch SCZ GWAS (464 cases and 1,272 controls, genotyped using Illumina's HumanHap550v3) [19] and a GWAS on the Danish sub-sample of the Scandinavian Collaboration on Psychiatric Etiology (573 cases and 453 controls, genotyped using Illumina's Human610-Quad, [44]).

#### GWAS of non-psychiatric phenotypes

As a control for the specificity of our analyses on cognitive traits and psychiatric illnesses, we also analysed non-psychiatric phenotypes. We performed GSEA on the non-psychiatric GWAS data sets from the WTCCC: Crohn's disease (1,748 cases), coronary heart disease (1,926 cases), hypertension (1,952 cases), rheumatoid arthritis (1,860 cases), type 1 diabetes (1,963 cases) and type 2 diabetes (1,924 cases). The GWAS data sets from the WTCCC included 2,938 healthy controls common for the six disorders. The individuals were genotyped using Affymetrix GC500K [42].

## Methods

### SNP to gene assignment using LDsnpR

In order to analyse the GWAS data on cognition, psychiatric disorders and non-psychiatric phenotypes at the gene level, we implemented a novel linkage disequilibrium (LD)-based SNP binning tool, named LDsnpR (Christoforou *et al.* under revision). This tool assigns SNP marker information and *P*-values from GWAS data sets to individual genes based both on the chromosomal position of the SNP and on the LD profile of the SNP (positional- and LD-based-binning, respectively). Thus, a SNP is assigned, or binned, to a gene if it is physically located within the pre-defined boundaries of the gene, or if it is in LD with another SNP (genotyped or not) that is physically located within these boundaries of the gene. Gene bin definitions were based on Human Ensembl release 54 (May 2009). They were further extended 10 kb on either side to best capture potential regulatory regions. The LD data was based on that of the CEU (CEPH (Utah residents with ancestry from northern and western Europe)) sample from HapMap Phase II release 27. The pairwise LD threshold was set at r^2^≥0.8.

### Gene scoring

The genes were scored with the minimum *P*-value observed among all the SNPs within each “gene bin”, adjusted for the number of SNPs assigned to each gene with a modified version of Sidak's correction [45], as implemented in LDsnpR. This method has been shown to perform as well as a powerful regression-based method in correcting for the bias due to SNP number [46]. Furthermore, we performed PLINK's permutation-based set method [47] on an *in house* data set and demonstrated a high correlation between the modified Sidak's corrected *P*-values and the permutation based *P*-values (r^2^>0.95, data not shown).

Results from gene- and gene set-based analysis, using raw unadjusted (for SNP number) minimum *P*-values, are provided in Tables S3, S4, and S5.

### Gene Set Enrichment Analysis

The 62 FMCx-, TCx- or OCx- genes were analysed as gene sets for enrichment of association signal in the GWAS data sets on cognition, psychiatric- and non-psychiatric phenotypes, using GSEA [32]. As described above, the GWAS SNPs were assigned to “gene bins” and scored using the modified Sidak's *P*-values. The genes were organised into ranked lists, upon which the gene sets were queried.

**Figure 1:**
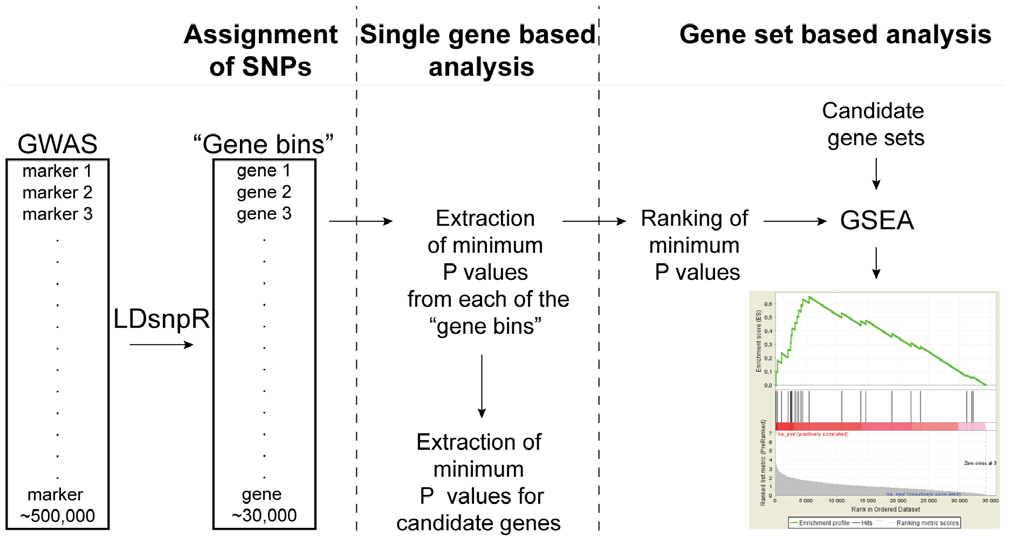
Schematic overview of the method. SNP markers from GWAS data were assigned to single genes in a process termed “gene binning”, by implementing a novel LD-based tool (LDsnpR, Christoforou *et al.* under revision). Modified Sidak's *P*-values were extracted for each gene (“gene bin”) in the GWAS data sets. Single gene-based analysis of the differentially expressed cortical genes was performed by extracting the modified Sidak's *P*-values for the candidate genes from the NCNG GWAS. Gene set-based analysis of the differentially expressed cortical genes was performed by extraction of the modified Sidak's *P*-values, followed by GSEA of GWAS data on cognition, psychiatric disorders and non-psychiatric phenotypes. GSEA: Gene set enrichment analysis, GWAS: Genome-wide association study.

The candidate genes were treated as four separate gene sets. Gene set 1: All cortex region enriched genes (FMCx, TCx and OCx, n = 62), Gene set 2: FMCx enriched genes (n = 29), Gene set 3: TCx enriched genes (n = 22) and Gene set 4: OCx enriched genes (n = 11) (Table 1–3). The GSEA 2.0 programme (http://www.broadinstitute.org/gsea/index.jsp) [32] was used to analyse the distribution of the candidate genes in the pre-ranked lists of genes from the different GWAS data sets. The gene sets were analysed in the ranked files, using weighted enrichment statistics (p = 1) and 1,500 permutations. The analysis was repeated three times to ensure consistency of results, and the false discovery rate (FDR) q-values were extracted for each trait/GWAS. See Figure 1 for schematic overview of the different steps in the procedure.

### Assessment of significance threshold

#### Gene Scores and multiple-testing correction

All reported gene-based *P*-values are uncorrected for the multiple psychometric traits and genes tested. Multiple testing correction in such a study is not straightforward, particularly due to the correlated nature of the test performed and the increased prior evidence supporting the relevance of these tests. However, a threshold corrected for these tests was determined as follows: Nine psychometric traits were tested in the NCNG sample. These traits are highly correlated, as shown in Table S2. Matrix Spectral Decomposition (matSpD; http://gump.qimr.edu.au/general/daleN/matSpD/) was applied to determine the equivalent number of independent traits tested, using the pairwise correlations between the traits [48]–[51]. V_effLi_ was estimated to be six, resulting in a Sidak-corrected threshold of 0.0085 required to keep the type 1 error rate at 5%. We further adjusted this threshold conservatively to account for the 62 genes tested, resulting in an experiment-wide threshold of 0.00014.

### GSEA

We employed three approaches to assess the validity and significance of our findings. First, we tested and compared with the GWASs of the six non-psychiatric phenotypes in the WTCCC [42]. Second, in addition to the cortical gene sets, we included a gene set consisting of various “housekeeping genes”, testing it across all cognitive, psychiatric- and non-psychiatric phenotypes (TaqMan endogenous controls from Applied Biosystems and a set of genes from Warrington *et al.* [52], Gene set 5: Housekeeping genes, n = 36, Table S6). Finally, for the significant gene sets, we ran the GSEA on 100 random gene sets. The random gene sets were generated using a pseudorandom number generator, randomly selecting genes from the Ensembl 54 definition. They were designed to mimic the significant gene sets, both with respect to the number of genes and the number of GWAS SNPs assigned to the genes (i.e. by LDsnpR) making up the gene set.

## Results

### Regionally enriched cortical genes show association to cognitive abilities

Based on our initial study of regional enrichment of genes in different parts of the rat neocortex, 62 genes were selected as candidates (Table 1–3) to search for association to nine different neurocognitive traits in the NCNG GWAS data set, covering four major cognitive domains: intellectual function, memory, executive attention and attention (Table S1). We took a candidate gene-based approach to the analysis, using a novel tool, LDsnpR, to assign SNPs to single genes based on chromosomal position and LD. LDsnpR was further used to score the genes, using the minimum *P*-value approach, adjusted for the number of SNPs in the gene “bins” with a modified Sidak's correction [45].

Several of the candidate genes displayed significant association to test measures of cognitive functions at the nominal, uncorrected significance level of 0.05 (Table 4–6, Table S7a–c), but none at the experiment-wide threshold of 0.00014. The overall strongest association in the analysis was observed between the TCx enriched gene *RAR-related orphan receptor B* (*RORB*) and the measure of verbal intelligence (Vocabulary, modified Sidak's *P* = 7.7E-04). In addition the FMCx enriched gene *Huntingtin-associated protein 1* (*HAP1*) displayed strong association to the measure of verbal intelligence (Vocabulary, modified Sidak's *P* = 8.9E-04) and nominal association to the full-scale measure of intellectual function (FSIQ, modified Sidak's *P* = 0.033). We also observed that three of the candidate genes showed nominal association to all the tests of attention (i.e. *Complement component 1, q subcomponent-like 3* (*C1QL3*), *Hypocretin (orexin) receptor 1* (*HCRTR1*) and *Calcium binding protein 1* (*CABP1*)).

**Table 4:** Gene-based analysis of frontomedial cortex enriched genes for association to cognitive abilities.

HGNC Symbol	SNPs	Intellectual function			Memory		Executive attention	Attention		
		FSIQ	Vocabulary	Reasoning	CVLT-L	CVLT-DR	Stroop3	CDT-Valid	CDT-Invalid	CDT-Neutral
ADPRHL1	13	0.0158	-	0.0422	-	-	-	-	-	-
ADRA1B	19	-	-	-	-	-	0.0456	-	-	-
ALDH3B2	8	-	-	-	-	-	-	-	-	-
C1QL3	16	-	-	-	0.0312	-	-	0.0021	0.0087	0.0022
CRIM1	81	-	-	-	-	-	-	-	-	-
CRIP2	2	-	-	-	-	-	-	-	-	-
EFNB3	5	-	-	-	-	-	-	-	-	-
EPHB6	13	-	-	-	-	-	-	-	-	-
FXYD6	25	-	-	-	-	-	-	-	-	-
GRP	14	-	-	-	-	-	-	-	-	-
HAP1	8	0.0326	**8.9E-04**	-	-	-	-	-	-	-
HCRTR1	11	-	-	-	-	-	-	0.0111	0.0074	0.0070
HEBP1	21	-	-	-	-	-	-	-	-	-
CADM1	70	-	-	-	-	-	-	-	0.0356	-
LDB2	129	-	-	-	-	-	-	-	-	-
LMO4	7	-	-	-	-	-	-	-	-	-
NAGS	5	-	-	-	-	-	-	-	-	-
NTF3	13	-	-	-	-	-	-	-	-	-
PANX1	22	-	-	-	-	-	-	-	-	-
PCDH17	33	-	-	-	-	-	-	-	-	-
PFKL	15	-	-	-	-	-	-	-	-	-
PRKCDBP	9	-	-	-	-	0.0466	-	-	-	-
PRMT2	16	-	-	-	-	-	-	-	-	-
RSPO2	54	-	-	-	-	-	-	-	-	-
RYR1	30	-	-	-	-	-	-	-	-	-
ST6GALNAC5	30	-	-	0.0269	-	-	-	-	-	-
SULF2	70	-	-	-	-	-	-	-	-	-
TMEFF1	27	-	-	-	-	-	-	-	-	-
ZCCHC12	4	-	-	-	-	-	-	-	-	-

The frontomedial cortex enriched genes (n = 29) were analysed for allelic association to nine test measures from the NCNG GWAS: **FSIQ**: estimated Full-Scale Intelligence Quotient, **Vocabulary**: Wechsler Abbreviated Scale of Intelligence, Vocabulary, **Reasoning**: Wechsler Abbreviated Scale of Intelligence, Matrix Reasoning, **CVLT-L**: California Verbal Learning Test, Learning measure, **CVLT-DR**: California Verbal Learning Test, Delayed free Recall, **Stroop3**: the third condition from the D-KEFS Color-Word Interference Test, **CDT**: Cued Discrimination Task, **Valid, Invalid and Neutral** [37]–[40]. The modified Sidak's minimum *P*-value for each candidate gene was extracted [45]. Only modified Sidak's *P*-values<0.05 are reported. “-”: non-significant *P*-value (i.e. *P*-values>0.05), HGNC: HUGO Gene Nomenclature Committee, SNPs: number of SNPs assigned to each gene by LDsnpR.

**Table 5 pone-0031687-t005:** Gene-based analysis of temporal cortex enriched genes for association to cognitive abilities.

HGNC Symbol	SNPs	Intellectual function			Memory		Executive attention	Attention		
		FSIQ	Vocabulary	Reasoning	CVLT-L	CVLT-DR	Stroop3	CDT-Valid	CDT-Invalid	CDT-Neutral
ARHGAP9	9	-	-	-	-	-	-	-	-	-
ATOH7	10	-	-	-	-	-	-	-	-	-
CA4	12	-	-	-	-	-	0.0071	-	-	-
CABP1	17	-	-	-	-	-	-	0.0193	0.0182	0.0408
CADPS2	91	-	-	-	-	-	-	-	-	-
COL13A1	106	-	-	-	-	-	-	-	-	-
GPR88	11	-	-	-	-	-	-	-	-	-
HHATL	12	-	-	-	-	-	-	-	-	-
IKBKE	20	-	-	-	-	-	-	-	-	-
JDP2	23	-	-	-	-	-	-	-	-	-
KCNC1	14	-	-	-	-	-	-	-	-	-
KCNS1	18	-	-	-	-	-	-	-	-	-
PLK5P	7	-	-	-	-	-	-	-	-	-
LPHN2	190	-	-	0.0273	-	-	-	-	-	-
LXN	15	-	-	-	-	-	0.0132	-	-	-
CD200R1	11	-	-	-	-	-	0.0335	-	-	-
NEFM	10	-	-	0.0056	-	-	-	-	-	-
NEU2	11	-	-	-	-	-	-	-	-	-
C1orf146	15	-	-	-	-	-	-	-	-	-
RORB	49	-	**7.7E-04**	-	-	-	0.0397	-	-	-
SCN1A	32	-	-	-	-	-	-	-	-	-
SCN4B	18	-	-	-	-	-	-	-	-	-

The temporal cortex enriched genes (n = 22) were analysed for allelic association to nine test measures from the NCNG GWAS. For trait abbreviations see Table 4. Modified Sidak's minimum *P*-value for each candidate gene was extracted [45], and only modified Sidak's *P*-values<0.05 are reported. “-”: non-significant *P*-value (i.e. *P*-values>0.05), HGNC: HUGO Gene Nomenclature Committee, SNPs: number of SNPs assigned to each gene by LDsnpR.

**Table 6:** Gene-based analysis of occipital cortex enriched genes for association to cognitive abilities.

HGNC Symbol	SNPs	Intellectual function			Memory		Executive attention	Attention		
		FSIQ	Vocabulary	Reasoning	CVLT-L	CVLT-DR	Stroop3	CDT-Valid	CDT-Invalid	CDT-Neutral
DCN	16	-	-	-	-	-	-	0.0087	-	0.0365
GPR68	9	-	0.0449	-	0.0111	-	-	-	-	-
HTR5B	33	-	-	-	-	-	-	-	-	-
HTRA4	7	-	-	-	-	-	-	-	-	-
IL12A	20	-	-	-	-	-	-	-	-	-
IRF6	14	-	-	-	-	-	-	-	-	-
KLF5	11	-	-	-	0.0226	-	-	-	-	-
MAB21L1	13	-	-	0.0110	-	-	-	-	-	-
NR2F1	7	-	-	-	-	-	-	-	-	-
ODZ3	161	0.0486	-	-	-	0.0328	-	-	-	-
SATB1	22	-	-	-	-	-	-	-	-	-

The occipital cortex enriched genes (n = 11) were analysed for allelic association to nine test measures from the NCNG GWAS. For trait abbreviations see Table 4. Modified Sidak's minimum *P*-value for each candidate gene was extracted [45], and only modified Sidak's *P*-values<0.05 are reported. “-”: non-significant *P*-value (i.e. *P*-values>0.05), HGNC: HUGO Gene Nomenclature Committee, SNPs: number of SNPs assigned to each gene by LDsnpR.

### Genes with preferential expression in the temporal cortex show enrichment of association signal to the Reasoning performance in GSEA

Next, we performed GSEA, to test the candidate genes for enrichment of association signal in test measures of cognitive functions. GSEA was originally developed to analyse the distribution of genes identified from microarray experiments, but has recently been implemented in the analysis of GWAS [32], [53].

We divided the candidate genes into gene sets based on their observed regional differences in expression (one set including all the differentially expressed cortical genes regardless of region and three gene sets composed of the genes enriched in the FMCx, TCx or OCx). In addition, we included a gene set comprising various “housekeeping” genes (from Applied Biosystems list of TaqMan endogenous controls and from Warrington *et al.* [52]). In order to test whether the candidate gene sets would show an overall enrichment for association to the nine cognitive test scores (Table S1), we used the “gene bins” and their assigned modified Sidak's *P*-values generated by LDsnpR as described above (see Method section for details and Figure 1).

We found that the TCx gene set showed significant enrichment of association signal to a test measure of non-verbal intelligence (Reasoning, FDR q-value = 0.06, cut-off FDR q-value set to 0.1, Table 7, Figure S1). The gene set comprised of “housekeeping” genes, used as a control for the specificity of our analysis, did not show significant enrichment to any of the neurocognitive tests. Furthermore, in order to validate the observed enrichment of association signal of the TCx genes (n = 22) in the test measure of non-verbal intelligence, 100 random gene sets were generated. Each of the hundred random gene sets comprised 22 arbitrary genes, each gene containing the same number of SNPs assigned to them, as the genes in the TCx gene set (see Methods for further details). Each random gene set was analysed using GSEA in the Reasoning GWAS, employing the same analysis statistics as applied for the TCx gene set. None of the random gene sets displayed significant enrichment of association signal (FDR q-values ranging from 0.52 to 1.0, for FDR q-value details see Table S8). This finding supports the robustness of the enrichment of association signal observed for the set of TCx genes to the test of non-verbal intelligence (Reasoning).

**Table 7:** GSEA of differentially expressed cortical genes in neurocognitive traits.

		All Cortex Regions (62)	Frontomedial Cortex (29)	Temporal Cortex (22)	Occipital Cortex (11)	Housekeeping genes (36)
**Intellectual function**	**FSIQ**	0.95	0.91	1.00	0.84	0.77
	**Vocabulary**	0.64	1.00	0.68	0.99	0.61
	**Reasoning**	0.18	0.56	**0.06**	0.16	0.73
**Executive attention**	**Stroop3**	0.76	0.87	0.59	0.93	0.52
**Memory**	**CVLT-L**	0.24	0.38	0.31	0.11	0.27
	**CVLT-DR**	0.84	0.95	0.62	0.86	0.82
**Attention**	**CDT-Valid**	0.31	0.44	0.33	0.27	0.63
	**CDT-Invalid**	0.52	0.52	0.39	**0.04**	0.52
	**CDT-Neutral**	0.36	0.30	0.18	0.16	0.29

The differentially expressed cortical genes were analysed as gene sets for enrichment of association signal in nine traits from the NCNG GWAS data [37]–[40], using GSEA [32]. Five gene sets were analysed; Gene set 1: combined list of all differentially expressed cortical genes, n = 62, Gene set 2: FMCx genes, n = 29, Gene set 3: TCx genes, n = 22, Gene set 4: OCx genes, n = 11, and Gene set 5: “housekeeping” genes, n = 36 (control gene set, Table S6). The analysis was based on extraction of modified Sidak's minimum *P*-values [45], as implemented in LDsnpR. FDR q-value<0.1 was set as cut-off value for significant enrichment. For trait abbreviations see Table 4.

We also observed an enrichment of association signal for the gene set comprising genes differentially expressed in the OCx and a test measure of attention (CDT-Invalid, FDR q-value 0.04, Table 7, Figure S1). Again, neither of the random gene sets mimicking the OCx gene set showed enrichment of association signal in the CDT-Invalid GWAS (FDR q-values ranging from 0.14 to 1.0, for FDR q-value details see Table S8), suggesting a role for genes expressed in the OCx in performance of an attention task.

### GSEA of genes differentially expressed in the frontomedial, temporal and occipital cortex in GWAS data of psychiatric disorders and non-psychiatric phenotypes

Since cognitive impairment constitutes a major endophenotype in patients suffering from SCZ and BP, and several cortical regions have been linked to disease susceptibility, we analysed the same gene sets by GSEA in three BP GWASs (the Norwegian TOP BP study, the British WTCCC BP and a German BP sample) and three SCZ GWASs (the Norwegian TOP SCZ study, a German SCZ sample and a Danish SCZ sample). In addition, we analysed six non-psychiatric phenotype GWASs from the WTCCC as controls (coronary heart disease, Crohn's Disease, hypertension, rheumatoid arthritis, type 1 diabetes and type 2 diabetes).

We found that the OCx gene set displayed enrichment of association signal to the Danish SCZ sample (FDR q-value 0.04, cut-off FDR q-value 0.1, Table 8, Figure S1). None of the cortical gene sets were enriched in the two other SCZ, nor in the three BP GWASs. When analysing the gene sets in the five non-psychiatric phenotype GWASs, no enrichment of association signal was observed (FDR q-value>0.1).

**Table 8:** GSEA of differentially expressed cortical genes in psychiatric disorders and non-psychiatric phenotypes.

	Origin of sample	All Cortex Regions (62)	Frontomedial Cortex (29)	Temporal Cortex (22)	Occipital Cortex (11)	Housekeeping genes (36)
**Bipolar Affective Disorder**	**TOP** *	0.26	0.24	0.26	0.87	0.81
	**German**	0.82	0.49	0.75	0.67	1.00
	**WTCCC** **	1.00	0.98	0.85	0.80	0.81
**Schizophrenia**	**TOP** *	0.70	0.73	0.80	0.63	0.85
	**German**	0.40	0.52	0.32	0.59	0.38
	**Danish**	0.71	0.61	0.79	**0.04**	0.70
**Non-psychiatric phenotypes, WTCCC**	**CD** **	0.65	0.75	0.50	1.00	1.00
	**CHD** **	1.00	1.00	0.87	0.87	1.00
	**HT** **	0.36	0.44	0.25	0.93	0.45
	**RA** **	0.29	0.36	0.26	0.21	0.27
	**T1D** **	0.68	0.89	0.84	1.00	1.00
	**T2D** **	0.11	0.20	0.10	0.16	0.21

GSEA was used to analyse the differentially expressed cortical genes, as gene sets, for enrichment of association signal in three different BP GWASs (a German sample, the Norwegian TOP sample and the British WTCCC BP sample [20], [41], [42]), three SCZ GWASs (the Norwegian TOP sample, the German part of a combined German-Dutch SCZ GWAS and a Danish sample [19], [43], [44]) and six non-psychiatric phenotypes (from WTCCC; CD: Crohn's disease, CHD: coronary heart disease, HT: hypertension, RA: rheumatoid arthritis, T1D: type 1 diabetes and T2D: type 2 diabetes, [42]). The analysis was based on extraction of modified Sidak's minimum *P*-values [45], as implemented in LDsnpR. FDR q-value<0.1 was set as cut-off value for significant enrichment.

* : One FMCx gene was not represented in the data set.

** : Two FMCx genes were not represented in the data set.

In this analysis, we also included a gene set consisting of “housekeeping” genes as a control for the specificity of our analysis. We did not observe any enrichment of association signal for this gene set in any of the psychiatric disorder or non-psychiatric phenotype GWASs analysed (FDR q-value>0.1). As a second control, GSEA was performed in the Danish SCZ GWAS using 100 random gene sets, consisting of 11 arbitrary genes (as previously described for the TCx and OCx gene sets in the test measures of reasoning and attention, respectively). None of the random gene sets displayed significant FDR q-values (FDR q-values ranging from 0.68 to 1.0, for FDR q-values details see Table S8). These findings support that the enrichment of association signal observed between the OCx gene set and the Danish SCZ GWAS was due to the genes contained in the OCx gene set, and not as a result of unspecific association signals.

### Functional annotation and gene expression patterns of the regionally enriched cortex genes in human

**Figure 2:**
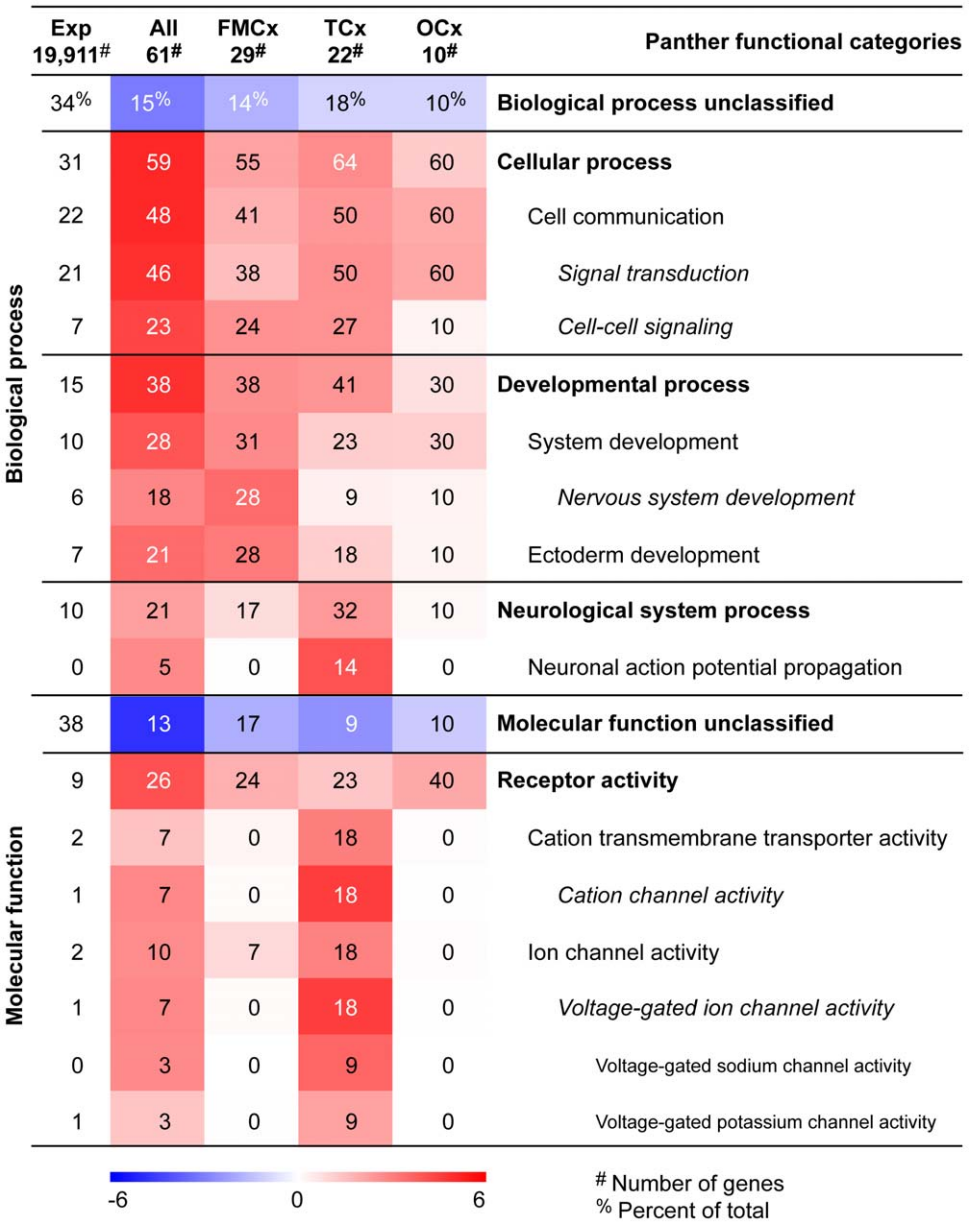
Functional characterisation of the human homologues to the rat regionally enriched cortical genes. Search for over- and under-represented biological processes and molecular functions was performed by using Panther [35], [36]. The significance of over- and under-represented Panther classification categories in the complete list of candidate genes (i.e. all the cortical regions, column 2), the FMCx enriched genes (column 3), TCx enriched genes (column 4) and OCx enriched genes (column 5), is illustrated by a heat map. The statistical significance of each gene set (negative log *P*-value) is illustrated by colour intensity (red: over-represented, blue: under-represented, white: as expected). Number of genes in each gene set is listed. The OCx gene *HTR5B* was not represented in Panther. The percentage of genes within a gene set that map to the given category is indicated on the heat map, e.g. 59% of the 61 enriched genes map to the biological process “cellular process”. The first column states the overall distribution of a term among the 19,911 genes from the default human reference gene list, e.g. 31% of the 61 regional genes were expected to map to ‘“cellular process”, hence this category is significantly over-represented among the regional genes. Exp: expected (based on default human reference gene list), FMCx: frontomedial cortex, TCx: temporal cortex, OCx: occipital cortex, #: number of genes in each gene set, %: percentage of genes.

The candidate genes analysed in this study were previously predicted to have a significant over-representation for particular biological processes and molecular functions in the rat, such as signal transduction, developmental processes and receptor activity [29]. In order to examine whether the candidate genes shared similar functional annotations in human, we mapped the entire set of regionally enriched genes, and in addition the gene sets composed of differentially expressed genes in the FMCx, TCx or OCx individually, to the Panther annotation categories. By comparing the distribution of the candidate genes to the human reference gene set provided (19,911 genes), we searched for significant over-representations of particular biological processes and molecular functions. Overall, the candidate genes were linked to cellular, developmental and neurological system processes (Figure 2). Furthermore, the candidate genes were found to be involved in receptor activity, primarily in cation transmembrane transporter activity and ion channel activity (Figure 2). Notably, the TCx gene set showed the strongest over-representation for most of the biological processes, and especially the molecular functional annotation, as compared to the FMCx and OCx gene sets.

We next analysed the expression pattern of a sub-set of the human homologues to the regionally enriched rat genes in the Allen Human Cortex Study (i.e. selected genes showing significant association in the NCNG sample). Although no quantitative differential gene expression could be detected, the homologous genes were expressed in corresponding regions in the human brain (e.g. FMCx, TCx or OCx enriched genes were expressed in the frontal, temporal or occipital lobe, respectively) (Figure S2A–C).

## Discussion

### Gene-based analysis of regionally enriched cortical genes for association to cognition

At the global level, the gene expression in different cortical regions is surprisingly similar, although highly specific functions are attributed to distinct cortical regions. Genes displaying differential expression in cortical regions might play an important role for the specialised normal function attributed to certain areas [29]. In this study, we used a novel set of differentially expressed cortical genes, identified from microarray gene expression profiling in the adult rat brain, to search for association at the single gene level to neurocognitive traits in human. In addition, we used a gene set-based approach to search for enrichment of association signal to cognitive traits and psychiatric disorders.

By mining GWAS data from a sample of healthy adults characterised by nine psychometric tests of cognitive function (the NCNG sample), and scoring the genes using LDsnpR, we found strong association between the TCx enriched gene *RORB* and a test of verbal intelligence (Vocabulary). This circadian clock gene has not previously been associated to cognitive abilities, but it is worth noting that the gene was recently ranked as one of the top candidate genes for susceptibility to BP in a large meta-analysis, and in a pediatric cohort of individuals suffering from BP [54], [55]. In the developing and adult rat brain, the gene is expressed in several regions associated with processing of sensory information, and behavioural changes (i.e. reduced anxiety and learned helplessness-related behaviour) have been observed in *Rorb* ^−/−^ mice [56], [57]. The *HAP1* gene also displayed a strong association to the measure of verbal intelligence (Vocabulary), and in addition, we observed a nominal association of *HAP1* to the estimated full-scale IQ (FSIQ). This gene has been shown to have an enriched expression in neurons, and the encoded protein is thought to be involved in intracellular trafficking and regulation of gene transcription. Dysfunction of *HAP1* has been linked to the neuropathology in Huntington disease, a disease where cognitive decline and psychiatric symptoms are often prominent (reviewed in [58]). Furthermore, we observed that the two FMCx enriched genes *C1QL3* and *HCRTR1*, and the TCx enriched gene *CABP1*, displayed significant association to all the tests of attention. Hcrtr1 has previously been shown to be involved in attentional processing by activating the basal forebrain cholinergic system in rats (reviewed in [59]). Interestingly, an association between *HCRTR1* and major mood disorders was recently reported [60]. Neither *C1QL3* nor *CABP1* have previously been linked to cognitive abilities. Notably, a reduction of neurons expressing *CABP1*, accompanied by an increase in protein expression in the remaining neurons, has been observed in post-mortem brain tissue from patients suffering from SCZ [61]. While none of these genes met the experiment-wide threshold of significance, *P* = 0.00014, which conservatively corrects for the number of traits and genes tested, these findings should be taken in the context of the prior evidence conferred on these candidate genes through the multiple relevant positive association, expression and functional results.

### Genes differentially expressed in the TCx show enrichment of association signal to a test measure of non-verbal intelligence in gene set-based analysis

In order to analyse whether the candidate genes as a group would show an association to cognitive traits, we chose to analyse them as gene sets, using GSEA in combination with the NCNG GWAS dataset. We found that the TCx gene set showed a significant enrichment of association signal to a test measure of non-verbal intelligence (Reasoning). In addition to analysing the gene set using modified Sidak's *P*-value, we also applied random gene sets that would mimic the TCx gene set in regard to number of genes contained in the set, and also SNP number assigned to each random gene. This analysis gave no significant enrichment of association signal, and it is therefore likely that the observed association is due to biological effects of the genes contained in the TCx gene set, and not as a result of unspecific association signal. We also included a gene set comprised of “housekeeping” genes in the analysis. This gene set showed no enrichment of association signal to any of the cognitive tests, further supporting the validity of the finding.

The parieto-frontal integration theory network, consisting of the dorsolateral prefrontal, parietal, anterior cingulate, temporal and occipital cortices, is suggested to explain differences in cognitive performances, including a test measure of reasoning [24]. The set of TCx genes analysed in this study, could be involved in this network, although the importance of the set of genes in intellectual function remains to be explored.

In the GSEA, we also observed an enrichment of association signal for the OCx gene set in one of the measures of attention (CDT-Invalid). The random gene sets used as a control gave no significant association, indicating that the observed enrichment was not a result of spurious association. However, the OCx gene set is fairly small (n = 11), and the finding could be a result of inflated scoring. The GSEA program estimates an enrichment score, and normalizes the score by taking the number of genes in the gene set into account. For very small gene sets (n<10), the probability of generating a false positive result will therefore increase, and caution has to be exercised with respect to the validity of this finding [32].

### Genes differentially expressed in the occipital cortex show enrichment of association signal to the Danish SCZ sample, in gene set-based analysis

Since impairments of cognitive functions are observed in individuals suffering from SCZ and BP, we also analysed the differentially expressed cortical genes, as gene sets, in GWASs of psychiatric illnesses, using GSEA.

We found that the OCx gene set displayed significant enrichment of association signal in the Danish SCZ GWAS. None of the cortical gene sets examined showed enrichment of association in the other SCZ, nor in the three BP GWASs analysed. In order to validate the findings, we generated 100 random gene sets mimicking the OCx gene set in regard to gene number and SNPs assigned to each gene. We did not observe an enrichment of association when analysing the random gene sets in GSEA, which could indicate that the observed association signal was due to the genes contained in the OCx gene set. In addition, we tested the validity of the GSEA in psychiatric disorder GWASs, by analysing the same candidate genes, as gene sets, in GWASs of non-psychiatric phenotypes from the WTCCC [42]. None of the gene sets showed enrichment of association signal. Furthermore, we also analysed a set of “housekeeping” genes in the six psychiatric disorders, and non-psychiatric phenotype data sets, and found no significant enrichment of association. Taken together the results could indicate an actual role for the genes contained in the OCx gene set in SCZ. On the other hand, the observed enrichment of association signal for the OCx gene set in the Danish SCZ GWAS was not observed in the other SCZ GWAS data sets examined. It is difficult to pinpoint the cause of this discrepancy. It is possible that it represents a false-positive finding. The OCx gene set comprised a small number of genes (n = 11), increasing the risk of generating a false positive result [32]. Alternatively, the genetic heterogeneity between the Norwegian, German and Danish populations might explain the observed differences [62]. This finding should anyway be considered with caution, and further replication studies are warranted.

### Regionally enriched cortical candidate genes; translation from rat to human

The candidate genes analysed in this study were identified from microarray gene expression profiling of the adult rat brain as differentially expressed genes in certain cortical regions. Despite the substantial difference in size, connectivity and cortical fields, some features of cortical organisation have been conserved in major groups of mammals [63], [64]. Areas within the OCx (i.e. primary and second visual areas), somatosensory areas and regions within the TCx (primary auditory area) are known to share common cortical fields in a large group of mammals [64]. The similarity in broad cortical field organisation is thought to be caused by genetic factors specifying regional identity, inherited from the common ancestor of all mammalian species [64]. Interestingly, a recent study showed that the genetically influenced cortical regionalisation in the human brain was similar to the regionalisation in rodents [65]. Furthermore, it has been demonstrated that the regional gene expression in the adult mouse anterior cortex, striatum and cerebellum showed very similar gene expression compared to the anatomically and functionally homologous human brain regions [66].

We found that the regionally enriched rat brain genes shared similar over-representations of functional annotations in human, as previously identified for the rat [29]. A sub-set of the human homologous genes were also found to be expressed in corresponding areas (i.e. human frontal, temporal or occipital lobes), as observed in the rat. Moreover, some of the candidate genes have previously been linked to psychiatric and neurological disorders (e.g. *RORB*, *HAP1*, *HCRTR1* and *CABP1*) [54], [55], [58], [60], [61], further emphasising the potential importance of these candidate genes in the human brain. On the other hand, some cortical areas are not well conserved in all mammals, e.g. the human frontal/prefrontal cortex, perisylvian cortex and the Broca's area (the site of speech generation). The prefrontal cortex is highly specialised in humans, being linked to higher order thinking, certain cognitive abilities and personality traits, whereas the frontomedial cortex from rat is mostly involved in motor functioning. It is therefore not surprising that we did not observe an enrichment of association signal to the FMCx gene set in GSEA.

Furthermore, the global gene expression in different cortical areas in human brain has been shown to vary more between individuals, than among regions within one individual [67]. Also, the inter-individual variation is apparently larger among humans than chimpanzees [67]. Rodents are a well established model system for studying human biology, given the ethical and practical limitations in using samples from the human brain. In rats, the variance in inter-individual gene expression is substantially less, and it therefore serves as a useful model for identifying differentially expressed genes in the adult neocortex.

### Conclusion

Our findings suggest an association between regionally enriched cortical genes and intellectual function. *RORB*, a promising candidate for susceptibility to BP, showed the overall strongest association in the analysis to a test of verbal intelligence. Moreover, we found that genes displaying preferential gene expression in the TCx showed enrichment of association signal to a test of non-verbal intelligence. We suggest that the TCx genes may be important to intellectual function in the healthy adult population. A replication of the findings is, however, essential to establish whether the TCx differentially expressed genes play a role in the neuronal mechanisms of intelligence.

## Supporting Information

Figure S1 **GSEA plots for gene sets displaying enrichment of association signal.** The upper part of the plots illustrates the running enrichment score for the gene sets, while the middle part of the plot illustrates the position of the individual genes (within the gene set) in the ranked list of genes. An accumulation of genes (black vertical lines) to the left on the x-axis indicate enrichment of association signal, reflected by the q-value (cut-off value<0.1, displayed in the upper right corner in each plot). The bottom part of the plots illustrates the value of the ranking metric. Upper panel: TCx gene set in a test measure of non-verbal intelligence (Reasoning). Middle panel: OCx gene set in a test measure of attention (CDT-Invalid). Lower panel: OCx gene set in the Danish SCZ. The corresponding GSEA plots for the “housekeeping” gene set are included.
(TIF)

Figure S2 **Cortical expression patterns of the human homologues to the regionally enriched rat genes.** The Whole Brain Microarray Survey in the Allen Human Cortex Study from the Allen Institute for Brain Science [34] was explored using Brain Explorer 2, in order to analyse the gene expression pattern of a sub-set of the candidate genes (i.e. selected genes showing significant association in the NCNG). Each sample from the microarray survey had been mapped to a 3D illustration of the MR picture of the donors (two donors in total). Orientation of the donor brains are indicated above the panels and also in the upper right part of each expression analysis picture. The left and middle panels illustrate the gene expression in either the frontal (**A**), temporal (**B**) or occipital (**C**) lobe, only. The right panel illustrates the overall gene expression in the cortex (all cortical regions selected). Red or green colour indicates high or low relative gene expression, respectively, compared to the different samples/structures in the brain. The human homologues to the rat genes were expressed in corresponding regions in the human brain (e.g. FMCx, TCx or OCx enriched genes were expressed in the frontal (**A**), temporal (**B**) or occipital (**C**) lobe, respectively).
(TIF)

Table S1 **Psychometric tests in the NCNG sample.** The individuals included in the NCNG sample underwent a battery of psychometric tests. The main references for the nine different tests focused on in the present study are listed.
(DOC)

Table S2 **Correlation between the psychometric tests in the NCNG.** Correlation estimates for the nine cognitive tests in the NCNG sample. For trait abbreviations see Table S1 and S3.
(DOC)

Table S3 **Gene-based analysis of regionally enriched cortical genes for association to cognitive abilities using uncorrected minimum** ***P*** **-values.** The cortical enriched genes were analysed for allelic association to nine tests from the NCNG GWAS [37]–[40]: **FSIQ**: estimated Full-Scale Intelligence Quotient, **Vocabulary**: Wechsler Abbreviated Scale of Intelligence, Vocabulary, **Reasoning**: Wechsler Abbreviated Scale of Intelligence, Matrix Reasoning, **CVLT-L**: California Verbal Learning Test, learning measure, **CVLT-DR**: California Verbal Learning Test, Delayed free Recall, **Stroop3**: the third condition from the D-KEFS Color-Word Interference Test, **CDT**: Cued Discrimination Task, **Valid, Invalid and Neutral**. The minimum *P*-value for each candidate gene was extracted, without adjusting for the number of SNPs assigned. Only uncorrected minimum *P*-values<0.05 are reported. “-”: non-significant *P*-value, HGNC: HUGO Gene Nomenclature Committee, SNPs: number of SNPs assigned to each gene by LDsnpR. *Table S3a: Frontomedial cortex enriched genes*, n = 29, *Table S3b: Temporal cortex enriched genes*, n = 22, and *Table S3c: Occipital cortex enriched genes*, n = 11.
(DOC)

Table S4 **GSEA of differentially expressed cortical genes in neurocognitive traits using uncorrected minimum** ***P*** **-values.** The differentially expressed cortical genes were analysed, as gene sets, for enrichment of association signal in nine tests measures of cognitive functions [37]–[40] from the NCNG GWAS data, using GSEA [32]. Five gene sets were analysed: gene set 1: combined list of all differentially expressed cortical genes, n = 62, gene set 2: FMCx genes, n = 29, gene set 3: TCx genes, n = 22, gene set 4: OCx genes, n = 11, and gene set 5: “housekeeping” genes, n = 36 (control gene set, Table S6). The analysis was based on extraction of minimum *P*-values, without correcting for the number of SNPs assigned to each gene in the GWAS data sets. FDR q-value<0.01 was set as cut-off value for significant enrichment. “*”: Nominal *P*-value<0.0006 (1/number of permutations (1,500) in the analysis). For trait abbreviations see Table S1 and S3.
(DOC)

Table S5 **GSEA of differentially expressed cortical genes in psychiatric disorders and non-psychiatric phenotypes using uncorrected minimum** ***P*** **-values.** GSEA was used to analyse the regionally enriched cortical genes, as gene-sets, for enrichment of association signal in three different BP GWASs (German, TOP and WTCCC [20], [41], [42]), three SCZ GWASs (the German part of a combined German-Dutch SCZ GWAS, TOP and a Danish SCZ sample [19], [43], [44]) and six non-psychiatric phenotypes (from WTCCC; CD: Crohn's disease, HT: hypertension, RA: rheumatoid arthritis, CHD: coronary heart disease, T1D: type 1 diabetes and T2D: type 2 diabetes [42]). The analysis was based on extraction of minimum *P*-values, without correcting for the number of SNPs assigned to each gene in the GWAS data sets. FDR q-value<0.01 was set as cut-off value for significant enrichment. The GSEA was performed 3 times, using 1,500 permutations and weighted enrichment statistics. Each run gave a slightly different FDR q-value, and the range for significant results are listed: a: (0.0020–0.0046), b: (0.013–0.021), c: (0.0088–0.014). *: One FMCx gene was not represented in the data set. **: Two FMCx genes were not represented in the data set.
(DOC)

Table S6 **Housekeeping genes used as a control gene set in GSEA.** HGNC symbol, Ensembl ID (Release 54) and description of housekeeping genes (n = 36) used as a control gene set in GSEA of the cognitive tests, psychiatric disorders and non-psychiatric phenotypes. The genes are from Applied Biosystem's list of TaqMan endogenous controls and from a list of housekeeping genes from Warrington *et al.* [52].
(DOC)

Table S7 **Gene based analysis of regionally enriched cortical genes for association to cognitive abilities (corrected).** The cortical enriched genes were analysed for allelic association to nine traits [37]–[40] from the NCNG GWAS. All modified Sidak's *P*-values are listed. HGNC: HUGO Gene Nomenclature Committee, SNPs: number of SNPs assigned to each gene by LDsnpR. For trait abbreviations see Table S1 and S3. *Table S7a*: Frontomedial cortex enriched genes, n = 29, *Table S7b*: Temporal cortex enriched genes, n = 22, and *Table S7c*: Occipital cortex enriched genes, n = 11.
(DOC)

Table S8 **Validation of observed enrichment by random gene sets.** The validity of the observed enrichment signal of the TCx gene set in the test measure of non-verbal intelligence (Reasoning), and the OCx gene set in both one of the attention tasks (CDT-Invalid) and the Danish SCZ sample, were analysed using random gene sets mimicking the gene sets in respect to gene set size and SNP markers assigned to each gene in the gene set. The ten best q-values are reported. RGS: Random Gene Set. FDR: False Discovery Rate.
(DOC)

## References

[1] Deary IJ, Penke L, Johnson W (2010). The neuroscience of human intelligence differences.. Nature Reviews Neuroscience.

[2] Deary IJ, Johnson W, Houlihan LM (2009). Genetic foundations of human intelligence.. Hum Genet.

[3] Davies G, Tenesa A, Payton A, Yang J, Harris SE (2011). Genome-wide association studies establish that human intelligence is highly heritable and polygenic.. Molecular psychiatry.

[4] Tsai SJ, Hong CJ, Yu YW, Chen TJ (2004). Association study of a brain-derived neurotrophic factor (BDNF) Val66Met polymorphism and personality trait and intelligence in healthy young females.. Neuropsychobiology.

[5] Winterer G, Goldman D (2003). Genetics of human prefrontal function.. Brain Res Brain Res Rev.

[6] Plomin R, Turic DM, Hill L, Turic DE, Stephens M (2004). A functional polymorphism in the succinate-semialdehyde dehydrogenase (aldehyde dehydrogenase 5 family, member A1) gene is associated with cognitive ability.. Mol Psychiatry.

[7] Deary IJ, Whiteman MC, Pattie A, Starr JM, Hayward C (2002). Cognitive change and the APOE epsilon 4 allele.. Nature.

[8] Le Hellard S, Havik B, Espeseth T, Breilid H, Lovlie R (2009). Variants in doublecortin- and calmodulin kinase like 1, a gene up-regulated by BDNF, are associated with memory and general cognitive abilities.. PLoS One.

[9] Gray JR, Thompson PM (2004). Neurobiology of intelligence: science and ethics.. Nature reviews Neuroscience.

[10] Sullivan PF, Kendler KS, Neale MC (2003). Schizophrenia as a complex trait: evidence from a meta-analysis of twin studies.. Arch Gen Psychiatry.

[11] Lichtenstein P, Bjork C, Hultman CM, Scolnick E, Sklar P (2006). Recurrence risks for schizophrenia in a Swedish national cohort.. Psychol Med.

[12] Dean K, Stevens H, Mortensen PB, Murray RM, Walsh E (2010). Full spectrum of psychiatric outcomes among offspring with parental history of mental disorder.. Arch Gen Psychiatry.

[13] Grozeva D, Kirov G, Ivanov D, Jones IR, Jones L (2010). Rare copy number variants: a point of rarity in genetic risk for bipolar disorder and schizophrenia.. Arch Gen Psychiatry.

[14] Owen MJ, Williams HJ, O'Donovan MC (2009). Schizophrenia genetics: advancing on two fronts.. Curr Opin Genet Dev.

[15] Shi J, Levinson DF, Duan J, Sanders AR, Zheng Y (2009). Common variants on chromosome 6p22.1 are associated with schizophrenia.. Nature.

[16] The International Schizophrenia Consortium (2009). Common polygenic variation contributes to risk of schizophrenia and bipolar disorder.. Nature.

[17] Stefansson H, Ophoff RA, Steinberg S, Andreassen OA, Cichon S (2009). Common variants conferring risk of schizophrenia.. Nature.

[18] Esslinger C, Walter H, Kirsch P, Erk S, Schnell K (2009). Neural mechanisms of a genome-wide supported psychosis variant.. Science.

[19] Rietschel M, Mattheisen M, Degenhardt F, Kahn RS, Linszen DH (2011). Association between genetic variation in a region on chromosome 11 and schizophrenia in large samples from Europe.. Molecular psychiatry.

[20] Cichon S, Muhleisen TW, Degenhardt FA, Mattheisen M, Miro X (2011). Genome-wide Association Study Identifies Genetic Variation in Neurocan as a Susceptibility Factor for Bipolar Disorder.. Am J Hum Genet.

[21] Ferreira MA, O'Donovan MC, Meng YA, Jones IR, Ruderfer DM (2008). Collaborative genome-wide association analysis supports a role for ANK3 and CACNA1C in bipolar disorder.. Nature genetics.

[22] O'Donovan MC, Craddock N, Norton N, Williams H, Peirce T (2008). Identification of loci associated with schizophrenia by genome-wide association and follow-up.. Nature genetics.

[23] Chubb JE, Bradshaw NJ, Soares DC, Porteous DJ, Millar JK (2008). The DISC locus in psychiatric illness.. Molecular psychiatry.

[24] Jung RE, Haier RJ (2007). The Parieto-Frontal Integration Theory (P-FIT) of intelligence: converging neuroimaging evidence.. The Behavioral and brain sciences.

[25] Rimol LM, Hartberg CB, Nesvag R, Fennema-Notestine C, Hagler DJ (2010). Cortical thickness and subcortical volumes in schizophrenia and bipolar disorder.. Biological psychiatry.

[26] Posthuma D, De Geus EJ, Baare WF, Hulshoff Pol HE, Kahn RS (2002). The association between brain volume and intelligence is of genetic origin.. Nat Neurosci.

[27] Shaw P, Greenstein D, Lerch J, Clasen L, Lenroot R (2006). Intellectual ability and cortical development in children and adolescents.. Nature.

[28] Stansberg C, Vik-Mo AO, Holdhus R, Breilid H, Srebro B (2007). Gene expression profiles in rat brain disclose CNS signature genes and regional patterns of functional specialisation.. BMC Genomics.

[29] Stansberg C, Ersland KM, van der Valk P, Steen VM (2011). Gene expression in the rat brain: High similarity but unique differences between frontomedial-, temporal- and occipital cortex.. BMC Neurosci.

[30] Roth RB, Hevezi P, Lee J, Willhite D, Lechner SM (2006). Gene expression analyses reveal molecular relationships among 20 regions of the human CNS.. Neurogenetics.

[31] Zhang W, Morris QD, Chang R, Shai O, Bakowski MA (2004). The functional landscape of mouse gene expression.. J Biol.

[32] Subramanian A, Tamayo P, Mootha VK, Mukherjee S, Ebert BL (2005). Gene set enrichment analysis: a knowledge-based approach for interpreting genome-wide expression profiles.. Proc Natl Acad Sci U S A.

[33] Flicek P, Aken BL, Ballester B, Beal K, Bragin E (2010). Ensembl's 10th year.. Nucleic acids research.

[34] Lein ES, Hawrylycz MJ, Ao N, Ayres M, Bensinger A (2007). Genome-wide atlas of gene expression in the adult mouse brain.. Nature.

[35] Thomas PD, Kejariwal A, Campbell MJ, Mi H, Diemer K (2003). PANTHER: a browsable database of gene products organized by biological function, using curated protein family and subfamily classification.. Nucleic acids research.

[36] Mi H, Dong Q, Muruganujan A, Gaudet P, Lewis S (2010). PANTHER version 7: improved phylogenetic trees, orthologs and collaboration with the Gene Ontology Consortium.. Nucleic acids research.

[37] Wechsler D (1999). Weschler abbreviated scale of intelligence.

[38] Delis DC, Karmer JH, Kaplan E, Ober BA (2000). California Verbal Learning Test.

[39] Delis DC, Kaplan E, Kramer JH (2001). D-KEFS: Examiners Manual.

[40] Espeseth T, Greenwood PM, Reinvang I, Fjell AM, Walhovd KB (2006). Interactive effects of APOE and CHRNA4 on attention and white matter volume in healthy middle-aged and older adults.. Cognitive, affective & behavioral neuroscience.

[41] Djurovic S, Gustafsson O, Mattingsdal M, Athanasiu L, Bjella T (2010). A genome-wide association study of bipolar disorder in Norwegian individuals, followed by replication in Icelandic sample.. J Affect Disord.

[42] WTCCC (2007). Genome-wide association study of 14,000 cases of seven common diseases and 3,000 shared controls.. Nature.

[43] Athanasiu L, Mattingsdal M, Kahler AK, Brown A, Gustafsson O (2010). Gene variants associated with schizophrenia in a Norwegian genome-wide study are replicated in a large European cohort.. J Psychiatr Res.

[44] Ingason A, Rujescu D, Cichon S, Sigurdsson E, Sigmundsson T (2011). Copy number variations of chromosome 16p13.1 region associated with schizophrenia.. Molecular psychiatry.

[45] Saccone SF, Hinrichs AL, Saccone NL, Chase GA, Konvicka K (2007). Cholinergic nicotinic receptor genes implicated in a nicotine dependence association study targeting 348 candidate genes with 3713 SNPs.. Human molecular genetics.

[46] Segre AV, Groop L, Mootha VK, Daly MJ, Altshuler D (2010). Common inherited variation in mitochondrial genes is not enriched for associations with type 2 diabetes or related glycemic traits.. PLoS genetics.

[47] Purcell S, Neale B, Todd-Brown K, Thomas L, Ferreira MA (2007). PLINK: a tool set for whole-genome association and population-based linkage analyses.. American journal of human genetics.

[48] Cheverud JM (2001). A simple correction for multiple comparisons in interval mapping genome scans.. Heredity.

[49] Li J, Ji L (2005). Adjusting multiple testing in multilocus analyses using the eigenvalues of a correlation matrix.. Heredity.

[50] Nyholt DR (2004). A simple correction for multiple testing for single-nucleotide polymorphisms in linkage disequilibrium with each other.. American journal of human genetics.

[51] The R Project for Statistical Computing website.. http://www.R-project.org.

[52] Warrington JA, Nair A, Mahadevappa M, Tsyganskaya M (2000). Comparison of human adult and fetal expression and identification of 535 housekeeping/maintenance genes.. Physiol Genomics.

[53] Wang K, Li M, Bucan M (2007). Pathway-based approaches for analysis of genomewide association studies.. American journal of human genetics.

[54] Le-Niculescu H, Patel SD, Bhat M, Kuczenski R, Faraone SV (2009). Convergent functional genomics of genome-wide association data for bipolar disorder: comprehensive identification of candidate genes, pathways and mechanisms.. Am J Med Genet B Neuropsychiatr Genet.

[55] McGrath CL, Glatt SJ, Sklar P, Le-Niculescu H, Kuczenski R (2009). Evidence for genetic association of RORB with bipolar disorder.. BMC Psychiatry.

[56] Masana MI, Sumaya IC, Becker-Andre M, Dubocovich ML (2007). Behavioral characterization and modulation of circadian rhythms by light and melatonin in C3H/HeN mice homozygous for the RORbeta knockout.. Am J Physiol Regul Integr Comp Physiol.

[57] Schaeren-Wiemers N, Andre E, Kapfhammer JP, Becker-Andre M (1997). The expression pattern of the orphan nuclear receptor RORbeta in the developing and adult rat nervous system suggests a role in the processing of sensory information and in circadian rhythm.. The European journal of neuroscience.

[58] Wu LL, Zhou XF (2009). Huntingtin associated protein 1 and its functions.. Cell adhesion & migration.

[59] Fadel J, Burk JA (2010). Orexin/hypocretin modulation of the basal forebrain cholinergic system: Role in attention.. Brain research.

[60] Rainero I, Ostacoli L, Rubino E, Gallone S, Picci LR (2011). Association between major mood disorders and the hypocretin receptor 1 gene.. Journal of affective disorders.

[61] Bernstein HG, Sahin J, Smalla KH, Gundelfinger ED, Bogerts B (2007). A reduced number of cortical neurons show increased Caldendrin protein levels in chronic schizophrenia.. Schizophrenia research.

[62] Lao O, Lu TT, Nothnagel M, Junge O, Freitag-Wolf S (2008). Correlation between genetic and geographic structure in Europe.. Current biology: CB.

[63] Krubitzer L (1995). The organization of neocortex in mammals: are species differences really so different?. Trends in neurosciences.

[64] Krubitzer L (2007). The magnificent compromise: cortical field evolution in mammals.. Neuron.

[65] Chen CH, Panizzon MS, Eyler LT, Jernigan TL, Thompson W (2011). Genetic influences on cortical regionalization in the human brain.. Neuron.

[66] Strand AD, Aragaki AK, Baquet ZC, Hodges A, Cunningham P (2007). Conservation of regional gene expression in mouse and human brain.. PLoS genetics.

[67] Khaitovich P, Muetzel B, She X, Lachmann M, Hellmann I (2004). Regional patterns of gene expression in human and chimpanzee brains.. Genome research.

