# Supplementary figures and images for: Gene-Based Analysis of Regionally Enriched Cortical Genes in GWAS Data Sets of Cognitive Traits and Psychiatric Disorders
Source: PLoS One. 2012 Feb 22;7(2):e31687. doi: 10.1371/journal.pone.0031687 (PMC3285182; doi:10.1371/journal.pone.0031687)

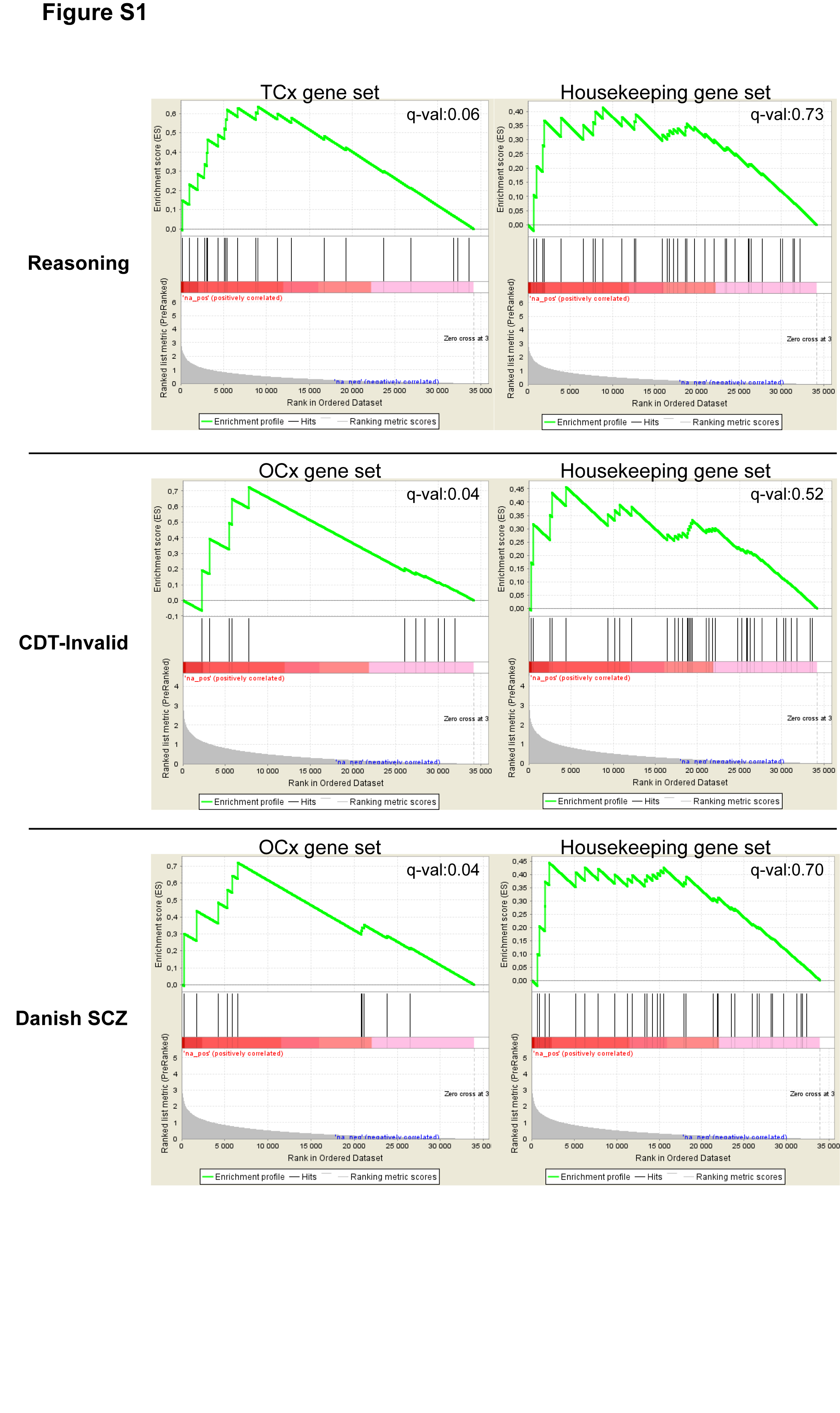

Supplement: Figure S1 — GSEA plots for gene sets displaying enrichment of association signal. The upper part of the plots illustrates the running enrichment score for the gene sets, while the middle part of the plot illustrates the position of the individual genes (within the gene set) in the ranked list of genes. An accumulation of genes (black vertical lines) to the left on the x-axis indicate enrichment of association signal, reflected by the q-value (cut-off value<0.1, displayed in the upper right corner in each plot). The bottom part of the plots illustrates the value of the ranking metric. Upper panel: TCx gene set in a test measure of non-verbal intelligence (Reasoning). Middle panel: OCx gene set in a test measure of attention (CDT-Invalid). Lower panel: OCx gene set in the Danish SCZ. The corresponding GSEA plots for the “housekeeping” gene set are included. (TIF) [file pone.0031687.s001.tif]

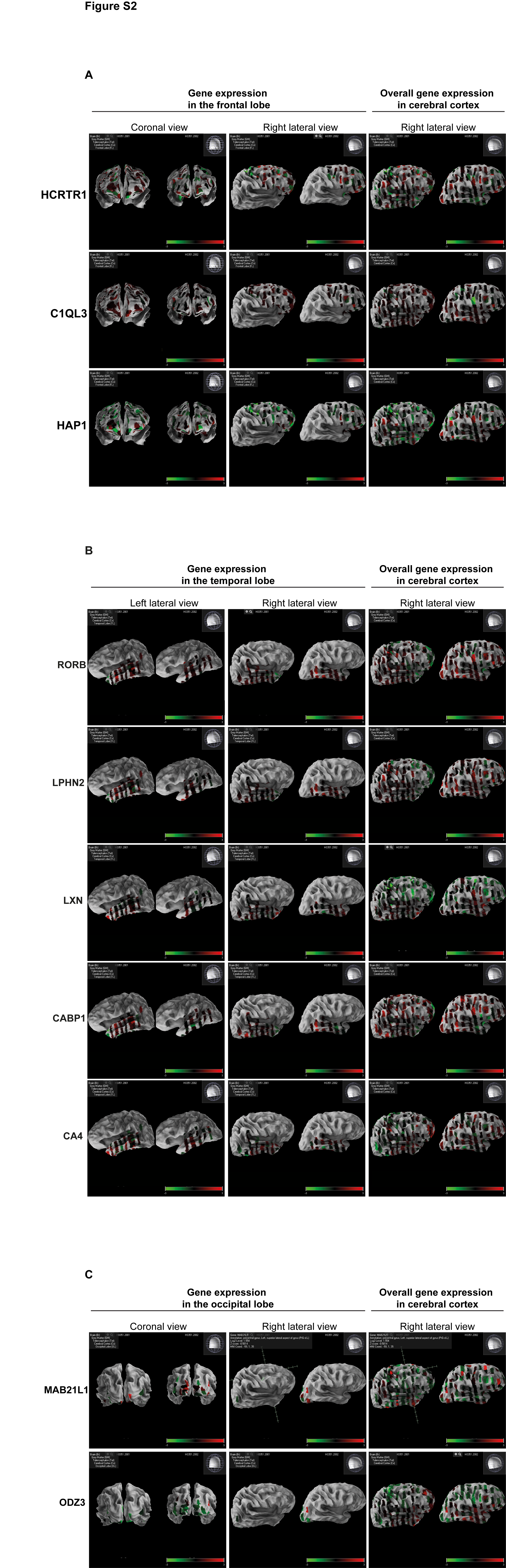

Supplement: Figure S2 — Cortical expression patterns of the human homologues to the regionally enriched rat genes. The Whole Brain Microarray Survey in the Allen Human Cortex Study from the Allen Institute for Brain Science [34] was explored using Brain Explorer 2, in order to analyse the gene expression pattern of a sub-set of the candidate genes (i.e. selected genes showing significant association in the NCNG). Each sample from the microarray survey had been mapped to a 3D illustration of the MR picture of the donors (two donors in total). Orientation of the donor brains are indicated above the panels and also in the upper right part of each expression analysis picture. The left and middle panels illustrate the gene expression in either the frontal (A), temporal (B) or occipital (C) lobe, only. The right panel illustrates the overall gene expression in the cortex (all cortical regions selected). Red or green colour indicates high or low relative gene expression, respectively, compared to the different samples/structures in the brain. The human homologues to the rat genes were expressed in corresponding regions in the human brain (e.g. FMCx, TCx or OCx enriched genes were expressed in the frontal (A), temporal (B) or occipital (C) lobe, respectively). (TIF) [file pone.0031687.s002.tif]
