# Supplementary material for: Gene-Based Analysis of Regionally Enriched Cortical Genes in GWAS Data Sets of Cognitive Traits and Psychiatric Disorders
Source: PLoS One. 2012 Feb 22;7(2):e31687. doi: 10.1371/journal.pone.0031687 (PMC3285182; doi:10.1371/journal.pone.0031687)
Supplement: Table S1 — Psychometric tests in the NCNG sample. The individuals included in the NCNG sample underwent a battery of psychometric tests. The main references for the nine different tests focused on in the present study are listed. (DOC) [file pone.0031687.s003.doc]

| **Table S1: Psychometric tests in the NCNG sample** | | | |
| --- | --- | --- | --- |
|  | **Test Name** | **Description** | **Reference** |
| **Intellectual function** | FSIQ | Full-Scale Intelligence Quotient | Wechsler *et al.* 1999 [37] |
|  | Vocabulary | Wechsler Abbreviated Scale of Intelligence, Vocabulary |  |
|  | Reasoning | Wechsler Abbreviated Scale of Intelligence, Matrix Reasoning |  |
| **Memory** | CVLT-L | California Verbal Learning Test, Learning measure | Delis *et al.* 2000 [38] |
|  | CVLT-DR | California Verbal Learning Test, Delayed free Recall |  |
| **Executive attention** | Stroop3 | D-KEFS Color-Word Interference, third condition | Delis *et al.* 2001 [39] |
| **Attention** | CDT-Valid | Cued Discrimination Task, Valid | Espeseth *et al.* 2006 [40] |
|  | CDT-Invalid | Cued Discrimination Task, Invalid |  |
|  | CDT-Neutral | Cued Discrimination Task, Neutral |  |
