# Supplementary material for: Gene-Based Analysis of Regionally Enriched Cortical Genes in GWAS Data Sets of Cognitive Traits and Psychiatric Disorders
Source: PLoS One. 2012 Feb 22;7(2):e31687. doi: 10.1371/journal.pone.0031687 (PMC3285182; doi:10.1371/journal.pone.0031687)
Supplement: Table S2 — Correlation between the psychometric tests in the NCNG. Correlation estimates for the nine cognitive tests in the NCNG sample. For trait abbreviations see Table S1 and S3. (DOC) [file pone.0031687.s004.doc]

| **Table S2: Correlation between the psychometric tests in the NCNG** | | | | | | | | | | |
| --- | --- | --- | --- | --- | --- | --- | --- | --- | --- | --- |
|  |  | **FSIQ** | **Reasoning** | **Vocabulary** | **Stroop3** | **CVLT-L** | **CVLT-DR** | **CDT-Valid** | **CDT-Invalid** | **CDT-Neutral** |
| **Intellectual function** | **FSIQ** | - |  |  |  |  |  |  |  |  |
|  | **Reasoning** | **0,64** | - |  |  |  |  |  |  |  |
|  | **Vocabulary** | **0,80** | 0,25 | - |  |  |  |  |  |  |
| **Executive attention** | **Stroop3** | -0,16 | -0,39 | -0,10 | - |  |  |  |  |  |
| **Memory** | **CVLT-L** | 0,31 | 0,39 | 0,30 | -0,36 | - |  |  |  |  |
|  | **CVLT-DR** | 0,27 | 0,34 | 0,28 | -0,30 | **0,81** | - |  |  |  |
| **Attention** | **CDT-Valid** | -0,16 | -0,39 | -0,10 | 0,49 | -0,35 | -0,32 | - |  |  |
|  | **CDT-Invalid** | -0,14 | -0,39 | -0,08 | **0,50** | -0,33 | -0,31 | **0,97** | - |  |
|  | **CDT-Neutral** | -0,15 | -0,40 | -0,08 | **0,50** | -0,35 | -0,32 | **0,98** | **0,97** | - |
