# Supplementary material for: Gene-Based Analysis of Regionally Enriched Cortical Genes in GWAS Data Sets of Cognitive Traits and Psychiatric Disorders
Source: PLoS One. 2012 Feb 22;7(2):e31687. doi: 10.1371/journal.pone.0031687 (PMC3285182; doi:10.1371/journal.pone.0031687)
Supplement: Table S3 — Gene-based analysis of regionally enriched cortical genes for association to cognitive abilities using uncorrected minimum P -values. The cortical enriched genes were analysed for allelic association to nine tests from the NCNG GWAS [37]–[40]: FSIQ: estimated Full-Scale Intelligence Quotient, Vocabulary: Wechsler Abbreviated Scale of Intelligence, Vocabulary, Reasoning: Wechsler Abbreviated Scale of Intelligence, Matrix Reasoning, CVLT-L: California Verbal Learning Test, learning measure, CVLT-DR: California Verbal Learning Test, Delayed free Recall, Stroop3: the third condition from the D-KEFS Color-Word Interference Test, CDT: Cued Discrimination Task, Valid, Invalid and Neutral. The minimum P-value for each candidate gene was extracted, without adjusting for the number of SNPs assigned. Only uncorrected minimum P-values<0.05 are reported. “-”: non-significant P-value, HGNC: HUGO Gene Nomenclature Committee, SNPs: number of SNPs assigned to each gene by LDsnpR. Table S3a: Frontomedial cortex enriched genes, n = 29, Table S3b: Temporal cortex enriched genes, n = 22, and Table S3c: Occipital cortex enriched genes, n = 11. (DOC) [file pone.0031687.s005.doc]

| **Table S3: Gene-based analysis of regionally enriched cortical genes for association to cognitive abilities using uncorrected minimum *P-*values** | | | | | | | | | | |
| --- | --- | --- | --- | --- | --- | --- | --- | --- | --- | --- |
| **Table S3a: Frontomedial cortex enriched genes, n = 29** | | | | | | | | | | |
| **HGNC Symbol** | **SNPs** | **Intellectual function** | | | **Memory** | | **Executive attention** | **Attention** | | |
|  |  | **FSIQ** | **Vocabulary** | **Reasoning** | **CVLT-L** | **CVLT-DR** | **Stroop3** | **CDT-Valid** | **CDT-Invalid** | **CDT-Neutral** |
| ADPRHL1 | 13 | 0.0023 | 0.040 | 0.0061 | - | - | - | 0.023 | - | 0.021 |
| ADRA1B | 19 | - | - | 0.0084 | 0.025 | 0.027 | 0.0047 | - | - | - |
| ALDH3B2 | 8 | - | - | - | - | - | - | - | - | - |
| C1QL3 | 16 | - | - | 0.044 | 0.0037 | 0.018 | - | 2.5E-04 | 0.0010 | 2.5E-04 |
| CRIM1 | 81 | 0.014 | - | 0.019 | 0.0016 | 0.0051 | 0.0061 | 0.0039 | 0.014 | 0.0092 |
| CRIP2 | 2 | - | - | - | - | - | - | - | - | - |
| EFNB3 | 5 | - | - | 0.044 | - | - | - | - | - | - |
| EPHB6 | 13 | 0.020 | 0.040 | 0.016 | 0.011 | - | - | 0.031 | - | 0.031 |
| FXYD6 | 25 | 0.043 | 0.0091 | - | - | - | 0.019 | - | - | - |
| GRP | 14 | - | 0.013 | - | - | - | - | - | - | - |
| HAP1 | 8 | 0.0073 | 2.0E-04 | - | - | - | - | - | - | - |
| HCRTR1 | 11 | - | - | - | - | 0.020 | - | 0.0019 | 0.0012 | 0.0012 |
| HEBP1 | 21 | - | - | - | - | - | 0.013 | 0.0090 | 0.015 | 0.0078 |
| CADM1 | 70 | 0.014 | - | 0.0017 | 0.026 | 0.010 | 0.019 | 0.0025 | 0.0010 | 0.0030 |
| LDB2 | 129 | 0.0066 | 0.026 | 0.0075 | 0.0045 | 0.0062 | 0.041 | 0.0055 | 0.0068 | 0.0064 |
| LMO4 | 7 | - | 0.014 | - | - | - | - | - | - | - |
| NAGS | 5 | - | - | - | - | - | - | 0.035 | 0.044 | 0.030 |
| NTF3 | 13 | - | 0.041 | - | - | - | - | 0.049 | 0.048 | - |
| PANX1 | 22 | 0.039 | - | 0.033 | - | 0.041 | - | 0.043 | - | 0.033 |
| PCDH17 | 33 | - | - | - | - | - | 0.023 | - | - | - |
| PFKL | 15 | - | - | 0.035 | 0.024 | - | - | - | - | - |
| PRKCDBP | 9 | - | 0.038 | - | 0.021 | 0.0095 | - | - | - | - |
| PRMT2 | 16 | - | - | - | - | 0.016 | - | - | - | - |
| RSPO2 | 54 | - | 0.026 | 0.036 | - | 0.035 | - | - | - | - |
| RYR1 | 30 | 0.018 | 0.0072 | 0.033 | 0.045 | 0.019 | - | - | - | - |
| ST6GALNAC5 | 30 | 0.012 | - | 0.0018 | 0.041 | 0.022 | 0.017 | 0.026 | 0.046 | 0.047 |
| SULF2 | 70 | 0.037 | - | 0.019 | 0.0079 | 0.0031 | 0.016 | 0.018 | 0.026 | 0.0092 |
| TMEFF1 | 27 | - | 0,027 | - | 0,027 | 0,018 | - | - | - | - |
| ZCCHC12 | 4 | - | - | - | 0.029 | - | - | - | - | - |

| **Table S3b: Temporal cortex enriched genes, n = 22** | | | | | | | | | | |
| --- | --- | --- | --- | --- | --- | --- | --- | --- | --- | --- |
| **HGNC Symbol** | **SNPs** | **Intellectual function** | | | **Memory** | | **Executive attention** | **Attention** | | |
|  |  | **FSIQ** | **Vocabulary** | **Reasoning** | **CVLT-L** | **CVLT-DR** | **Stroop3** | **CDT-Valid** | **CDT-Invalid** | **CDT-Neutral** |
| ARHGAP9 | 9 | - | - | - | - | - | - | - | - | - |
| ATOH7 | 10 | - | - | - | - | - | - | - | - | - |
| CA4 | 12 | - | - | - | - | 0.0087 | 0.0011 | 0.034 | 0.010 | 0.015 |
| CABP1 | 17 | - | - | 0.016 | 0.019 | - | - | 0.0022 | 0.0020 | 0.0046 |
| CADPS2 | 91 | 0.0068 | - | 0.010 | - | 0.039 | 0.028 | - | 0.038 | - |
| COL13A1 | 106 | 0.0026 | 0.0058 | 0.0034 | 0.019 | 0.032 | 0.0070 | 0.0015 | 0.0024 | 0.0024 |
| GPR88 | 11 | 0.020 | - | 0.014 | - | - | 0.028 | - | - | - |
| HHATL | 12 | - | - | - | 0.0089 | 0.015 | 0.020 | 0.022 | 0.020 | 0.018 |
| IKBKE | 20 | 0.007 | - | 0.0094 | 0.028 | - | 0.0072 | 0.017 | 0.026 | 0.016 |
| JDP2 | 23 | 0.010 | - | 0.019 | - | 0.020 | - | - | - | - |
| KCNC1 | 14 | - | - | 0.0080 | - | - | 0.049 | 0.024 | 0.015 | 0.020 |
| KCNS1 | 18 | - | - | - | - | - | 0.049 | 0.035 | 0.038 | 0.015 |
| PLK5P | 7 | - | - | 0.025 | - | 0.013 | - | - | - | - |
| LPHN2 | 190 | 5.8E-04 | 0.0020 | 2.9E-04 | 6.5E-04 | 9.0E-04 | 0.0054 | 0.022 | 0.026 | 0.029 |
| LXN | 15 | - | - | - | - | - | 0.0017 | - | - | - |
| CD200R1 | 11 | - | 0.017 | - | - | - | 0.0057 | - | - | - |
| NEFM | 10 | 0.015 | - | 0.0010 | - | - | - | - | - | - |
| NEU2 | 11 | - | - | - | 0.011 | - | - | - | - | - |
| C1orf146 | 15 | - | 0.043 | - | - | - | - | - | - | - |
| RORB | 49 | 0.015 | 3.1E-05 | 0.013 | 0.0082 | 0.031 | 0.0016 | 0.044 | 0.048 | 0.044 |
| SCN1A | 32 | 0.028 | - | 0.011 | 0.023 | 0.036 | - | - | - | - |
| SCN4B | 18 | 0.012 | 0.019 | 0.018 | - | - | - | - | - | - |

| **Table S3c: Occipital cortex enriched genes, n = 11** | | | | | | | | | | |
| --- | --- | --- | --- | --- | --- | --- | --- | --- | --- | --- |
| **HGNC Symbol** | **SNPs** | **Intellectual function** | | | **Memory** | | **Executive attention** | **Attention** | | |
|  |  | **FSIQ** | **Vocabulary** | **Reasoning** | **CVLT-L** | **CVLT-DR** | **Stroop3** | **CDT-Valid** | **CDT-Invalid** | **CDT-Neutral** |
| DCN | 16 | - | - | - | 0.033 | - | 0.029 | 0.0010 | 0.0085 | 0.0044 |
| GPR68 | 9 | 0.041 | 0.0092 | - | 0.0022 | - | - | - | - | - |
| HTR5B | 33 | 0.048 | - | - | - | 0.029 | - | - | - | - |
| HTRA4 | 7 | - | - | - | - | - | - | - | - | - |
| IL12A | 20 | - | - | 0.0084 | - | - | - | - | - | - |
| IRF6 | 14 | 0.026 | 0.012 | - | 0.013 | - | 0.0078 | - | - | - |
| KLF5 | 11 | - | - | 0.042 | 0.0038 | 0.031 | - | 0.033 | 0.032 | 0.031 |
| MAB21L1 | 13 | 0.019 | 0.031 | 0.0016 | - | 0.048 | - | - | - | - |
| NR2F1 | 7 | - | - | - | - | - | - | - | - | - |
| ODZ3 | 161 | 6.2E-04 | 0.0077 | 0.035 | 0.0014 | 4.1E-04 | 0.0031 | 0.0021 | 0.0026 | 0.0029 |
| SATB1 | 22 | - | - | - | - | - | - | 0.0059 | 0.0090 | 0.012 |
