# Supplementary material for: Gene-Based Analysis of Regionally Enriched Cortical Genes in GWAS Data Sets of Cognitive Traits and Psychiatric Disorders
Source: PLoS One. 2012 Feb 22;7(2):e31687. doi: 10.1371/journal.pone.0031687 (PMC3285182; doi:10.1371/journal.pone.0031687)
Supplement: Table S4 — GSEA of differentially expressed cortical genes in neurocognitive traits using uncorrected minimum P -values. The differentially expressed cortical genes were analysed, as gene sets, for enrichment of association signal in nine tests measures of cognitive functions [37]–[40] from the NCNG GWAS data, using GSEA [32]. Five gene sets were analysed: gene set 1: combined list of all differentially expressed cortical genes, n = 62, gene set 2: FMCx genes, n = 29, gene set 3: TCx genes, n = 22, gene set 4: OCx genes, n = 11, and gene set 5: “housekeeping” genes, n = 36 (control gene set, Table S6). The analysis was based on extraction of minimum P-values, without correcting for the number of SNPs assigned to each gene in the GWAS data sets. FDR q-value<0.01 was set as cut-off value for significant enrichment. “*”: Nominal P-value<0.0006 (1/number of permutations (1,500) in the analysis). For trait abbreviations see Table S1 and S3. (DOC) [file pone.0031687.s006.doc]

| **Table S4: GSEA of differentially expressed cortical genes in neurocognitive traits using uncorrected minimum *P-*values** | | | | | | |
| --- | --- | --- | --- | --- | --- | --- |
|  |  | **All Cortex Regions (62)** | **Frontomedial Cortex (29)** | **Temporal Cortex (22)** | **Occipital Cortex (11)** | **Housekeeping genes (36)** |
| **Intellectual function** | **FSIQ** | 0.07 | 0.10 | 0.04 | 0.33 | 0.78 |
|  | **Vocabulary** | 0.33 | 0.25 | 0.44 | 0.31 | 0.88 |
|  | **Reasoning** | **0.01** | 0.08 | **0.00*** | 0.20 | 0.65 |
| **Executive attention** | **Stroop3** | 0.12 | 0.50 | 0.04 | 0.35 | 0.50 |
| **Memory** | **CVLT-L** | 0.23 | 0.14 | 0.13 | 0.10 | 0.43 |
|  | **CVLT-DR** | 0.08 | 0.09 | 0.10 | 0.16 | 0.92 |
| **Attention** | **CDT-Valid** | 0.10 | 0.19 | 0.21 | 0.11 | 0.92 |
|  | **CDT-Invalid** | 0.20 | 0.23 | 0.20 | 0.23 | 0.96 |
|  | **CDT-Neutral** | 0.13 | 0.10 | 0.19 | 0.30 | 0.77 |
