# Supplementary material for: Gene-Based Analysis of Regionally Enriched Cortical Genes in GWAS Data Sets of Cognitive Traits and Psychiatric Disorders
Source: PLoS One. 2012 Feb 22;7(2):e31687. doi: 10.1371/journal.pone.0031687 (PMC3285182; doi:10.1371/journal.pone.0031687)
Supplement: Table S5 — GSEA of differentially expressed cortical genes in psychiatric disorders and non-psychiatric phenotypes using uncorrected minimum P -values. GSEA was used to analyse the regionally enriched cortical genes, as gene-sets, for enrichment of association signal in three different BP GWASs (German, TOP and WTCCC [20], [41], [42]), three SCZ GWASs (the German part of a combined German-Dutch SCZ GWAS, TOP and a Danish SCZ sample [19], [43], [44]) and six non-psychiatric phenotypes (from WTCCC; CD: Crohn's disease, HT: hypertension, RA: rheumatoid arthritis, CHD: coronary heart disease, T1D: type 1 diabetes and T2D: type 2 diabetes [42]). The analysis was based on extraction of minimum P-values, without correcting for the number of SNPs assigned to each gene in the GWAS data sets. FDR q-value<0.01 was set as cut-off value for significant enrichment. The GSEA was performed 3 times, using 1,500 permutations and weighted enrichment statistics. Each run gave a slightly different FDR q-value, and the range for significant results are listed: a: (0.0020–0.0046), b: (0.013–0.021), c: (0.0088–0.014). *: One FMCx gene was not represented in the data set. **: Two FMCx genes were not represented in the data set. (DOC) [file pone.0031687.s007.doc]

| **Table S5: GSEA of differentially expressed cortical genes in psychiatric disorders and non-psychiatric phenotypes using uncorrected minimum *P-*values** | | | | | | |
| --- | --- | --- | --- | --- | --- | --- |
|  | **Origin of sample** | **All Cortex Regions (62)** | **Frontomedial Cortex (29)** | **Temporal Cortex (22)** | **Occipital Cortex (11)** | **Housekeeping genes (36)** |
| **Bipolar Affective Disorder** | **TOP*** | 0.02 | 0.24 | **0.0046 a** | 0.35 | 0.64 |
|  | **German** | 0.23 | 0.14 | 0.40 | 0.41 | 0.43 |
|  | **WTCCC**** | 0.54 | 0.90 | 0.48 | 0.77 | 0.97 |
| **Schizophrenia** | **TOP*** | 0.38 | 0.53 | 0.29 | 0.29 | 1.00 |
|  | **German** | 0,39 | 0,14 | 0,77 | 0,44 | 0,52 |
|  | **Danish** | 0.33 | 0.44 | 0.43 | 0.41 | 0.61 |
| **Non-psychiatric phenotypes, WTCCC** | **CD**** | 0.03 | 0.03 | 0.04 | 0.33 | 0.64 |
|  | **CHD**** | 0.13 | 0.12 | **0.013 b** | 0.82 | 0.24 |
|  | **HT**** | 0.02 | 0.03 | **0.014 c** | 0.69 | 0.74 |
|  | **RA**** | 0.10 | 0.21 | 0.24 | 0.23 | 0.54 |
|  | **T1D**** | 0.37 | 0.54 | 0.43 | 0.50 | 0.28 |
|  | **T2D**** | 0.17 | 0.17 | 0.18 | 0.16 | 0.88 |
