# Supplementary material for: Gene-Based Analysis of Regionally Enriched Cortical Genes in GWAS Data Sets of Cognitive Traits and Psychiatric Disorders
Source: PLoS One. 2012 Feb 22;7(2):e31687. doi: 10.1371/journal.pone.0031687 (PMC3285182; doi:10.1371/journal.pone.0031687)
Supplement: Table S6 — Housekeeping genes used as a control gene set in GSEA. HGNC symbol, Ensembl ID (Release 54) and description of housekeeping genes (n = 36) used as a control gene set in GSEA of the cognitive tests, psychiatric disorders and non-psychiatric phenotypes. The genes are from Applied Biosystem's list of TaqMan endogenous controls and from a list of housekeeping genes from Warrington et al. [52]. (DOC) [file pone.0031687.s008.doc]

| **Table S6: Housekeeping genes used as a control gene set in GSEA** | | |
| --- | --- | --- |
| **HGNC Symbol** | **Ensembl ID/54** | **Description** |
| ACTB | ENSG00000075624 | actin, beta |
| AGPAT1 | ENSG00000204310 | 1-acylglycerol-3-phosphate O-acyltransferase 1 (lysophosphatidic acid acyltransferase, alpha) |
| ATP5O | ENSG00000159186 | ATP synthase subunit O, mitochondrial Precursor (Oligomycin sensitivity conferral protein)(OSCP) |
| B2M | ENSG00000166710 | beta-2-microglobulin |
| BCAT2 | ENSG00000105552 | branched chain amino-acid transaminase 2, mitochondrial |
| CAPN2 | ENSG00000162909 | calpain 2, (m/II) large subunit |
| CETN2 | ENSG00000147400 | centrin, EF-hand protein, 2 |
| DGCR6 | ENSG00000183628 | DiGeorge syndrome critical region gene 6 |
| EIF2B2 | ENSG00000119718 | eukaryotic translation initiation factor 2B, subunit 2 beta, 39kDa |
| GUSB | ENSG00000169919 | glucuronidase, beta |
| HMBS | ENSG00000149397 | hydroxymethylbilane synthase |
| HPRT1 | ENSG00000165704 | hypoxanthine phosphoribosyltransferase 1 |
| HTR2C | ENSG00000147246 | 5-hydroxytryptamine (serotonin) receptor 2C |
| HYAL2 | ENSG00000068001 | hyaluronoglucosaminidase 2 |
| MLF2 | ENSG00000089693 | myeloid leukemia factor 2 |
| MPRIP | ENSG00000133030 | myosin phosphatase Rho interacting protein |
| PGK1 | ENSG00000102144 | phosphoglycerate kinase 1 |
| PMM1 | ENSG00000100417 | phosphomannomutase 1 |
| POLR2F | ENSG00000100142 | polymerase (RNA) II (DNA directed) polypeptide F |
| PPIB | ENSG00000166794 | peptidylprolyl isomerase B (cyclophilin B) |
| PPP1CC | ENSG00000186298 | protein phosphatase 1, catalytic subunit, gamma isozyme |
| PRDX6 | ENSG00000117592 | peroxiredoxin 6 |
| PSMA5 | ENSG00000143106 | Proteasome subunit alpha type-5 |
| [PSMD7](http://www.ensembl.org/Homo_sapiens/Gene/Summary?g=ENSG00000103035) | ENSG00000103035 | proteasome (prosome, macropain) 26S subunit, non-ATPase, 7 |
| RNPS1 | ENSG00000205937 | RNA binding protein S1, serine-rich domain |
| RPLP0P2 | ENSG00000089157 | [60S acidic ribosomal protein P0 (L10E)](http://www.uniprot.org/uniprot/?query=P05388&sort=score) |
| SIGMAR1 | ENSG00000147955 | sigma non-opioid intracellular receptor 1 |
| TAGLN2 | ENSG00000158710 | transgelin 2 |
| TAX1BP1 | ENSG00000106052 | Tax1 (human T-cell leukemia virus type I) binding protein 1 |
| TFRC | ENSG00000072274 | transferrin receptor (p90, CD71) |
| TTC1 | ENSG00000113312 | tetratricopeptide repeat domain 1 |
| TXNRD1 | ENSG00000198431 | thioredoxin reductase 1 |
| UBC | ENSG00000150991 | ubiquitin C |
| VIL2 | ENSG00000092820 | ezrin |
| WARS | ENSG00000140105 | tryptophanyl-tRNA synthetase |
| YWHAZ | ENSG00000164924 | tyrosine 3-monooxygenase/tryptophan 5-monooxygenase activation protein, zeta polypeptide |
|  |  |  |
