# Supplementary material for: Gene-Based Analysis of Regionally Enriched Cortical Genes in GWAS Data Sets of Cognitive Traits and Psychiatric Disorders
Source: PLoS One. 2012 Feb 22;7(2):e31687. doi: 10.1371/journal.pone.0031687 (PMC3285182; doi:10.1371/journal.pone.0031687)
Supplement: Table S7 — Gene based analysis of regionally enriched cortical genes for association to cognitive abilities (corrected). The cortical enriched genes were analysed for allelic association to nine traits [37]–[40] from the NCNG GWAS. All modified Sidak's P-values are listed. HGNC: HUGO Gene Nomenclature Committee, SNPs: number of SNPs assigned to each gene by LDsnpR. For trait abbreviations see Table S1 and S3. Table S7a: Frontomedial cortex enriched genes, n = 29, Table S7b: Temporal cortex enriched genes, n = 22, and Table S7c: Occipital cortex enriched genes, n = 11. (DOC) [file pone.0031687.s009.doc]

| **Table S7: Gene based analysis of regionally enriched cortical genes for association to cognitive abilities (corrected)** | | | | | | | | | | |
| --- | --- | --- | --- | --- | --- | --- | --- | --- | --- | --- |
| **Table S7a: Frontomedial cortex enriched genes, n = 29** | | | | | | | | | | |
| **HGNC Symbol** | **SNPs** | **Intellectual function** | | | **Memory** | | **Executive attention** | **Attention** | | |
|  |  | **FSIQ** | **Vocabulary** | **Reasoning** | **CVLT-L** | **CVLT-DR** | **Stroop3** | **Valid- RT** | **Invalid-RT** | **Neutral-RT** |
| ADPRHL1 | 13 | 0.0158 | 0.2500 | 0.0422 | 0.6212 | 0.6627 | 0.9746 | 0.1500 | 0.3924 | 0.1366 |
| ADRA1B | 19 | 0.4872 | 0.6463 | 0.0808 | 0.2199 | 0.2370 | 0.0456 | 0.4860 | 0.8104 | 0.7036 |
| ALDH3B2 | 8 | 0.5939 | 0.3758 | 0.9125 | 0.8111 | 0.8434 | 0.9891 | 0.8124 | 0.8462 | 0.8844 |
| C1QL3 | 16 | 0.7102 | 0.6672 | 0.3206 | 0.0312 | 0.1409 | 0.9403 | 0.0021 | 0.0087 | 0.0022 |
| CRIM1 | 81 | 0.4402 | 0.8995 | 0.5425 | 0.0653 | 0.1886 | 0.2222 | 0.1495 | 0.4501 | 0.3143 |
| CRIP2 | 2 | 0.1155 | 0.3250 | 0.2001 | 0.3440 | 0.8546 | 0.3708 | 0.5673 | 0.4795 | 0.4838 |
| EFNB3 | 5 | 0.2254 | 0.5172 | 0.1270 | 0.3294 | 0.4448 | 0.3155 | 0.9666 | 0.9463 | 0.9075 |
| EPHB6 | 13 | 0.1288 | 0.2458 | 0.1066 | 0.0738 | 0.9430 | 0.5418 | 0.1992 | 0.5952 | 0.1993 |
| FXYD6 | 25 | 0.4349 | 0.1116 | 0.9702 | 0.7207 | 0.8835 | 0.2194 | 0.5741 | 0.7856 | 0.6589 |
| GRP | 14 | 0.4177 | 0.0912 | 0.9093 | 0.7212 | 0.9712 | 0.3448 | 0.6742 | 0.5869 | 0.6574 |
| HAP1 | 8 | 0.0326 | 0.0009 | 0.2241 | 0.9875 | 0.8651 | 0.2273 | 0.5376 | 0.7368 | 0.4861 |
| HCRTR1 | 11 | 0.3495 | 0.5422 | 0.5136 | 0.4915 | 0.1116 | 0.9451 | 0.0111 | 0.0074 | 0.0070 |
| HEBP1 | 21 | 0.9907 | 0.8190 | 0.9988 | 0.9098 | 0.9395 | 0.1362 | 0.0949 | 0.1506 | 0.0826 |
| CADM1 | 70 | 0.3936 | 0.8571 | 0.0597 | 0.6112 | 0.3076 | 0.4955 | 0.0836 | 0.0356 | 0.1014 |
| LDB2 | 129 | 0.3484 | 0.8204 | 0.3850 | 0.2541 | 0.3326 | 0.9358 | 0.3026 | 0.3585 | 0.3430 |
| LMO4 | 7 | 0.5231 | 0.0536 | 0.4623 | 0.5297 | 0.7246 | 0.5000 | 0.4154 | 0.6957 | 0.2400 |
| NAGS | 5 | 0.9056 | 0.7263 | 0.5710 | 0.2816 | 0.2969 | 0.5880 | 0.1024 | 0.1271 | 0.0870 |
| NTF3 | 13 | 0.7273 | 0.2552 | 0.5298 | 0.4618 | 0.4504 | 0.9180 | 0.2947 | 0.2929 | 0.3898 |
| PANX1 | 22 | 0.3683 | 0.5563 | 0.3172 | 0.7168 | 0.3840 | 0.6893 | 0.3998 | 0.6420 | 0.3201 |
| PCDH17 | 33 | 0.6128 | 0.8144 | 0.8299 | 0.9628 | 0.6983 | 0.3301 | 0.9817 | 0.8581 | 0.9907 |
| PFKL | 15 | 0.9793 | 0.9735 | 0.2456 | 0.1766 | 0.3562 | 0.7139 | 0.8088 | 0.6328 | 0.8522 |
| PRKCDBP | 9 | 0.5174 | 0.1762 | 0.3220 | 0.1020 | 0.0466 | 0.6178 | 0.5380 | 0.4842 | 0.4381 |
| PRMT2 | 16 | 0.9819 | 0.8969 | 0.6691 | 0.6720 | 0.1246 | 0.5371 | 0.9309 | 0.9015 | 0.9465 |
| RSPO2 | 54 | 0.9383 | 0.5143 | 0.6301 | 0.8689 | 0.6201 | 0.9136 | 0.8186 | 0.7936 | 0.8448 |
| RYR1 | 30 | 0.2479 | 0.1059 | 0.4067 | 0.5097 | 0.2592 | 0.8814 | 0.9215 | 0.9799 | 0.9432 |
| ST6GALNAC5 | 30 | 0.1675 | 0.5875 | 0.0269 | 0.4778 | 0.2911 | 0.2283 | 0.3366 | 0.5191 | 0.5288 |
| SULF2 | 70 | 0.7369 | 0.9017 | 0.5012 | 0.2464 | 0.1053 | 0.4308 | 0.4769 | 0.6019 | 0.2786 |
| TMEFF1 | 27 | 0.7070 | 0.3211 | 0.7857 | 0.3199 | 0.2289 | 0.5978 | 0.8755 | 0.8281 | 0.6510 |
| ZCCHC12 | 4 | 0.6262 | 0.9460 | 0.3874 | 0.0712 | 0.2565 | 0.8400 | 0.3822 | 0.5680 | 0.5093 |

| **Table S7b: Temporal cortex enriched genes, n = 22** | | | | | | | | | | |
| --- | --- | --- | --- | --- | --- | --- | --- | --- | --- | --- |
| **HGNC Symbol** | **SNPs** | **Intellectual function** | | | **Memory** | | **Executive attention** | **Attention** | | |
|  |  | **FSIQ** | **Vocabulary** | **Reasoning** | **CVLT-L** | **CVLT-DR** | **Stroop3** | **Valid-RT** | **Invalid-RT** | **Neutral-RT** |
| ARHGAP9 | 9 | 0.8355 | 0.4305 | 0.9749 | 0.8138 | 0.9936 | 0.4543 | 0.4190 | 0.4422 | 0.5342 |
| ATOH7 | 10 | 0.9574 | 0.5837 | 0.2861 | 0.7539 | 0.9293 | 0.9614 | 0.5545 | 0.8652 | 0.5053 |
| CA4 | 12 | 0.9631 | 0.8445 | 0.6265 | 0.3197 | 0.0552 | 0.0071 | 0.1993 | 0.0616 | 0.0918 |
| CABP1 | 17 | 0.5147 | 0.6693 | 0.1340 | 0.1577 | 0.4450 | 0.9542 | 0.0193 | 0.0182 | 0.0408 |
| CADPS2 | 91 | 0.2703 | 0.9782 | 0.3637 | 0.9763 | 0.8425 | 0.7239 | 0.9470 | 0.8336 | 0.9451 |
| COL13A1 | 106 | 0.1296 | 0.2663 | 0.1650 | 0.6417 | 0.8283 | 0.3151 | 0.0790 | 0.1187 | 0.1220 |
| GPR88 | 11 | 0.1132 | 0.6661 | 0.0835 | 0.3598 | 0.5156 | 0.1543 | 0.9309 | 0.9359 | 0.8094 |
| HHATL | 12 | 0.7365 | 0.7689 | 0.9823 | 0.0566 | 0.0926 | 0.1210 | 0.1320 | 0.1202 | 0.1129 |
| IKBKE | 20 | 0.0695 | 0.6836 | 0.0940 | 0.2617 | 0.5970 | 0.0729 | 0.1688 | 0.2439 | 0.1534 |
| JDP2 | 23 | 0.1155 | 0.5404 | 0.2067 | 0.8319 | 0.2121 | 0.9970 | 0.9633 | 0.9398 | 0.9911 |
| KCNC1 | 14 | 0.6094 | 0.4848 | 0.0587 | 0.7341 | 0.4764 | 0.3138 | 0.1683 | 0.1089 | 0.1382 |
| KCNS1 | 18 | 0.9912 | 0.8126 | 0.8606 | 0.9839 | 0.9791 | 0.3824 | 0.2874 | 0.3103 | 0.1332 |
| PLK5P | 7 | 0.2783 | 0.3561 | 0.0952 | 0.3929 | 0.0507 | 0.4983 | 0.6047 | 0.2834 | 0.3907 |
| LPHN2 | 190 | 0.0537 | 0.1741 | 0.0273 | 0.0606 | 0.0821 | 0.4035 | 0.8858 | 0.9205 | 0.9417 |
| LXN | 15 | 0.6028 | 0.9980 | 0.5439 | 0.9963 | 0.5279 | 0.0132 | 0.6595 | 0.4776 | 0.5123 |
| CD200R1 | 11 | 0.3500 | 0.0984 | 0.7674 | 0.7674 | 0.7250 | 0.0335 | 0.5826 | 0.4325 | 0.6239 |
| NEFM | 10 | 0.0778 | 0.8343 | 0.0056 | 0.8009 | 0.5277 | 0.3656 | 0.9836 | 0.9374 | 0.9995 |
| NEU2 | 11 | 0.6005 | 0.5667 | 0.4171 | 0.0656 | 0.4554 | 0.8132 | 0.6439 | 0.2715 | 0.7319 |
| C1orf146 | 15 | 0.6504 | 0.2939 | 0.9981 | 0.9529 | 0.9983 | 0.8772 | 0.7371 | 0.8674 | 0.7791 |
| RORB | 49 | 0.3096 | 0.0008 | 0.2774 | 0.1869 | 0.5475 | 0.0397 | 0.6785 | 0.7057 | 0.6761 |
| SCN1A | 32 | 0.3717 | 0.9877 | 0.1628 | 0.3181 | 0.4583 | 0.7923 | 0.9929 | 0.9999 | 0.9894 |
| SCN4B | 18 | 0.1103 | 0.1680 | 0.1570 | 0.7028 | 0.5559 | 0.4016 | 0.9010 | 0.6569 | 0.9025 |

| **Table S7c: Occipital cortex enriched genes, n = 11** | | | | | | | | | | |
| --- | --- | --- | --- | --- | --- | --- | --- | --- | --- | --- |
| **HGNC Symbol** | **SNPs** | **Intellectual function** | | | **Memory** | | **Executive attention** | **Attention** | | |
|  |  | **FSIQ** | **Vocabulary** | **Reasoning** | **CVLT-L** | **CVLT-DR** | **Stroop3** | **Valid-RT** | **Invalid-RT** | **Neutral-RT** |
| SCN1A | 32 | 0.3717 | 0.9877 | 0.1628 | 0.3181 | 0.4583 | 0.7923 | 0.9929 | 0.9999 | 0.9894 |
| SCN4B | 18 | 0.1103 | 0.1680 | 0.1570 | 0.7028 | 0.5559 | 0.4016 | 0.9010 | 0.6569 | 0.9025 |
| DCN | 16 | 0.7510 | 0.7010 | 0.9437 | 0.2490 | 0.9595 | 0.2199 | 0.0087 | 0.0702 | 0.0365 |
| GPR68 | 9 | 0.1872 | 0.0449 | 0.4410 | 0.0111 | 0.2697 | 0.4267 | 0.4856 | 0.2540 | 0.2530 |
| HTR5B | 33 | 0.5654 | 0.7646 | 0.7564 | 0.7639 | 0.3961 | 0.9836 | 0.9160 | 0.9786 | 0.9945 |
| HTRA4 | 7 | 0.3358 | 0.2013 | 0.1990 | 0.9574 | 0.8250 | 0.8918 | 0.8703 | 0.9580 | 0.9476 |
| IL12A | 20 | 0.6329 | 0.8344 | 0.0847 | 0.9359 | 0.5612 | 0.4984 | 0.8632 | 0.9424 | 0.6945 |
| IRF6 | 14 | 0.1783 | 0.0863 | 0.6633 | 0.0962 | 0.3459 | 0.0568 | 0.4876 | 0.8464 | 0.4576 |
| KLF5 | 11 | 0.7024 | 0.3915 | 0.2264 | 0.0226 | 0.1713 | 0.4609 | 0.1815 | 0.1755 | 0.1740 |
| MAB21L1 | 13 | 0.1281 | 0.1956 | 0.0110 | 0.3889 | 0.2936 | 0.5342 | 0.7734 | 0.8816 | 0.7547 |
| NR2F1 | 7 | 0.5441 | 0.6671 | 0.2166 | 0.2436 | 0.5612 | 0.9807 | 0.7130 | 0.9101 | 0.8850 |
| ODZ3 | 161 | 0.0486 | 0.4652 | 0.9445 | 0.1107 | 0.0328 | 0.2220 | 0.1570 | 0.1883 | 0.2124 |
| SATB1 | 22 | 0.4988 | 0.6472 | 0.6014 | 0.9140 | 0.9160 | 0.7392 | 0.0653 | 0.0987 | 0.1272 |
