# Supplementary material for: Gene-Based Analysis of Regionally Enriched Cortical Genes in GWAS Data Sets of Cognitive Traits and Psychiatric Disorders
Source: PLoS One. 2012 Feb 22;7(2):e31687. doi: 10.1371/journal.pone.0031687 (PMC3285182; doi:10.1371/journal.pone.0031687)
Supplement: Table S8 — Validation of observed enrichment by random gene sets. The validity of the observed enrichment signal of the TCx gene set in the test measure of non-verbal intelligence (Reasoning), and the OCx gene set in both one of the attention tasks (CDT-Invalid) and the Danish SCZ sample, were analysed using random gene sets mimicking the gene sets in respect to gene set size and SNP markers assigned to each gene in the gene set. The ten best q-values are reported. RGS: Random Gene Set. FDR: False Discovery Rate. (DOC) [file pone.0031687.s010.doc]

| **Table S8: Validation of observed enrichment by random gene sets** | | | | | |
| --- | --- | --- | --- | --- | --- |
| **Reasoning** | | **CDT-Invalid** | | **Danish SCZ** | |
| **RGS** | **FDR q-val** | **RGS** | **FDR q-val** | **RGS** | **FDR q-val** |
| RGS36 | 0.52 | RGS35 | 0.14 | RGS49 | 0.68 |
| RGS25 | 0.53 | RGS74 | 0.86 | RGS22 | 0.79 |
| RGS60 | 0.53 | RGS16 | 0.88 | RGS18 | 0.82 |
| RGS18 | 0.57 | RGS59 | 0.90 | RGS33 | 0.82 |
| RGS76 | 0.60 | RGS91 | 0.90 | RGS17 | 0.82 |
| RGS14 | 0.64 | RGS27 | 0.90 | RGS51 | 0.82 |
| RGS44 | 0.67 | RGS83 | 0.90 | RGS43 | 0.83 |
| RGS10 | 0.67 | RGS52 | 0.90 | RGS62 | 0.83 |
| RGS11 | 0.68 | RGS1 | 0.91 | RGS7 | 0.83 |
| RGS1 | 0.69 | RGS6 | 0.91 | RGS29 | 0.83 |
